# *Cadherin‐3* is a novel oncogenic biomarker with prognostic value in glioblastoma

**DOI:** 10.1002/1878-0261.13162

**Published:** 2022-06-10

**Authors:** Eduarda P. Martins, Céline S. Gonçalves, Marta Pojo, Rita Carvalho, Ana S. Ribeiro, Vera Miranda‐Gonçalves, Ricardo Taipa, Fernando Pardal, Afonso A. Pinto, Carlos Custódia, Cláudia C. Faria, Fátima Baltazar, Nuno Sousa, Joana Paredes, Bruno M. Costa

**Affiliations:** ^1^ Life and Health Sciences Research Institute (ICVS) School of Medicine University of Minho Braga Portugal; ^2^ ICVS/3B’s ‐ PT Government Associate Laboratory Braga/Guimarães Portugal; ^3^ i3S – Instituto de Investigação e Inovação em Saúde Universidade do Porto Porto Portugal; ^4^ Neuropathology Unit Department of Neurosciences Centro Hospitalar do Porto Porto Portugal; ^5^ Department of Pathology Hospital de Braga Portugal; ^6^ Department of Neurosurgery, Hospital de Braga Portugal; ^7^ Instituto de Medicina Molecular Faculdade de Medicina Universidade de Lisboa Portugal; ^8^ Neurosurgery Department Hospital de Santa Maria Centro Hospitalar Lisboa Norte (CHLN) Portugal; ^9^ Faculty of Medicine of the University of Porto Portugal

**Keywords:** biomarker, *CDH3*/P‐cadherin, glioblastoma, survival, tumor aggressiveness

## Abstract

Glioblastoma (GBM) is the most common and malignant primary brain tumor in adults. The prognosis of patients is very poor, with a median overall survival of ~ 15 months after diagnosis. Cadherin‐3 (also known as P‐cadherin), a cell–cell adhesion molecule encoded by the *CDH3* gene, is deregulated in several cancer types, but its relevance in GBM is unknown. In this study, we investigated the functional roles, the associated molecular signatures, and the prognostic value of *CDH3*/P‐cadherin in this highly malignant brain tumor. *CDH3*/P‐cadherin mRNA and protein levels were evaluated in human glioma samples. Knockdown and overexpression models of P‐cadherin in GBM were used to evaluate its functional role *in vitro* and *in vivo*. *CDH3*‐associated gene signatures were identified by enrichment analyses and correlations. The impact of *CDH3* in the survival of GBM patients was assessed in independent cohorts using both univariable and multivariable models. We found that P‐cadherin protein is expressed in a subset of gliomas, with an increased percentage of positive samples in grade IV tumors. Concordantly, *CDH3* mRNA levels in glioma samples from The Cancer Genome Atlas (TCGA) database are increased in high‐grade gliomas. P‐cadherin displays oncogenic functions in multiple knockdown and overexpression GBM cell models by affecting cell viability, cell cycle, cell invasion, migration, and neurosphere formation capacity. Genes that were positively correlated with *CDH3* are enriched for oncogenic pathways commonly activated in GBM. *In vivo*, GBM cells expressing high levels of P‐cadherin generate larger subcutaneous tumors and cause shorter survival of mice in an orthotopic intracranial model. Concomitantly, high *CDH3* expression is predictive of shorter overall survival of GBM patients in independent cohorts. Together, our results show that *CDH3*/P‐cadherin expression is associated with aggressiveness features of GBM and poor patient prognosis, suggesting that it may be a novel therapeutic target for this deadly brain tumor.

## Graphical abstract

Here, we identified *Cadherin‐3 (CDH3)* as a novel oncogene in glioblastoma (GBM). *CDH3* affected distinct cancer hallmarks *in vitro* and was related to increased tumor growth and shorter survival *in vivo*. Clinically, *CDH3* correlated with cancer‐related signatures and was overexpressed in a subset of patients with poor prognosis, thus suggesting that *CDH3* could hold a prognostic value for patients with GBM (Scheme drawn with images from Servier Medical Art; https://smart.servier.com/).

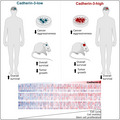

**Abbreviations**
CI: confidence interval
CNS: central nervous system
DGAV: Direção Geral de Alimentação e Veterinária
DMEM: Dulbecco’s modified Eagle’s medium
ES: Enrichment Score
FBS: fetal bovine serum
FDR: false discovery rate
FELASA: Federation of European Laboratory Animal Science Associations
FFPE: formalin‐fixed paraffin‐embedded
FPKM‐UQ: fragments per kilobase of transcript per Million mapped reads upper quartile
GBM: glioblastoma
GO: gene ontology
GSEA: gene set enrichment analysis
H&E: hematoxylin and eosin
HR: hazard ratio
IHC: immunohistochemistry
KEGG: Kyoto encyclopedia of genes and genomes
KPS: Karnofsky Performance Score
NSG: Nod *scid* gamma
NT: nontumor
OS: overall survival
PI: propidium iodide
qRT‐PCR: quantitative reverse transcription‐polymerase chain reaction
RPMI: Roswell Park Memorial Institute medium
RT: room temperature
TCGA: The Cancer Genome Atlas
WB: western blot
WHO: World Health Organization

## 1 Introduction

Gliomas represent a major portion of primary central nervous system (CNS) tumors, being glioblastoma (GBM) the most common and malignant form in adults [1]. Patients diagnosed with GBM present a median overall survival (OS) of approximately 15 months under standard‐of‐care treatment, based on tumor surgical resection and postoperative chemoradiotherapy [2, 3]. Despite many advances in our understanding of the cellular and molecular characteristics of GBM, treatments did not significantly improve over the last few decades [4]. More recently, a variety of immunotherapies [5] (e.g., with immune checkpoint inhibitors [6, 7]) and oncolytic virus therapies (e.g., with the recombinant oncolytic poliovirus PVSRIPO [8]) have emerged as potentially useful for GBM patients, but their value to improve overall survival is still limited. The identification of new molecular players relevant for GBM aggressiveness may allow a better stratification and understanding of this malignancy, potentially contributing to a better clinical management of patients.

Cadherins are cell–cell adhesion molecules dependent on calcium with a crucial role in tissue morphogenesis and maintenance of structural and functional tissues [9]. P(Placental)‐cadherin, encoded by the *CDH3* gene, is a type I classical cadherin, firstly identified in murine placenta [10]. In humans, P‐cadherin is also expressed in the placenta, but at lower levels than in mice, and is mainly found in basal layers of epithelial tissues [11, 12, 13]. In the last years, similarly to other classical cadherins, the relevance of P‐cadherin in various cancers has been highly documented [14, 15]. In breast cancer, P‐cadherin overexpression is associated with poor clinical outcome and tumor aggressiveness, associating with tumor stem cell properties and invasiveness capacity [16, 17, 18, 19]. Paradoxically, in malignant melanoma, P‐cadherin induces an anti‐invasive behavior by promoting cell–cell adhesion [20, 21], highlighting the dual oncogenic or tumor‐suppressor roles of P‐cadherin according to the tumor type and particular molecular context (reviewed in ref. [15]). Interestingly, the relevance of P‐cadherin in malignant gliomas was not previously studied.

In this work, using *in vitro* and *in vivo* models, as well as patient‐derived samples, we have established *CDH3*/P‐cadherin as a novel oncogenic player in human GBM, deciphering its functional roles, and as a novel prognostic biomarker predictive of shorter survival in GBM patients.

## 2 Materials and methods

### 2.1 Cell lines and cell culture

The human GBM cell lines U87MG and U373MG (kindly provided by Dr. Joseph Costello, University of California San Francisco, CA, USA), SNB19 (DSMZ, German Collection of Microorganisms and Cell Cultures, Braunschweig, Germany), U251MG, A172, LN229 (ATCC, American Type Culture Collection, Manassas, VA, USA), and the GBM patient‐derived cultures GBML18 (previously established in our group [22]), GBML24, GBML45, and GBML95 were cultured in Dulbecco’s modified Eagle’s medium (DMEM, Biochrom GmbH, Berlin, Germany), supplemented with 10% fetal bovine serum (FBS, Biochrom GmbH). The GBM patient‐derived culture GBML42 (established in our group, as previously described [23]) was maintained in Roswell Park Memorial Institute (RPMI, Biochrom GmbH) 1640 medium, supplemented with 10% FBS. Human embryonic kidney HEK293T cells (kindly provided by Dr. Andreia Neves‐Carvalho, ICVS/University of Minho, Portugal) used for lentiviral packaging were cultured in DMEM supplemented with 10% FBS. Complete medium is defined as DMEM or RPMI (according to the cell culture) supplemented with 10% FBS. All cells were maintained in a humidified atmosphere, with 5% (v/v) CO_2_ and at 37 °C, unless otherwise stated. GBM cell lines were authenticated by short tandem repeat profiling. All cultures were tested for mycoplasma contamination every month.

### 2.2 Glioma samples

Glioma tumors were obtained at Hospital de Braga (Braga, Portugal) and Hospital de Santa Maria (Lisbon, Portugal) during craniotomy surgeries for tumor resection or biopsy. Samples from both hospitals were used for mRNA expression levels evaluation. For that, samples were transported in dry ice from the Hospital to the laboratory and stored at −80 °C until further treatment. The samples from Hospital de Braga used for immunohistochemical analyses were formalin‐fixed and paraffin‐embedded.

### 2.3 *CDH3* overexpression in GBM cell line

U87MG cells were plated at an initial density of 2 × 10^5^ cells per well (in a 6‐well plate). On the next day, cells were transfected with the vector pIRES2‐EGFP‐CDH3 (U87‐CDH3) that contains full‐length cDNA encoding *CDH3*, or with the empty vector pIRES2‐EGFP (U87‐Ctrl) [24] using the lipofectamine 3000 transfection reagent (Invitrogen, Thermo Fisher Scientific, Waltham, MA, USA). Lipofectamine 3000 transfection reagent (4 µL per well) was diluted in Opti‐Modified Eagle Reduced Serum Medium (Opti‐MEM, Gibco, Thermo Fisher Scientific, Waltham, MA, USA), and the DNA mix was prepared by diluting the transfection vector (1 µg) in Opti‐MEM and adding P3000 reagent (2 µL per µg DNA). Afterward, diluted DNA was incubated with the diluted lipofectamine for 15 min at room temperature (RT) and added dropwise to the cells. Cells were incubated with the transfection medium for 48 h. Transfected cells were selected through the incubation with geneticin (G418, 800 µg·mL^−1^, Santa Cruz Biotechnology, Dallas, TX, USA) in complete medium.

### 2.4 *CDH3* silencing in GBM primary cultures by siRNA and shRNA

For the transfection with siRNAs, GBML18 cells were plated at an initial density of 8 × 10^5^ cells per T25 cm^2^ culture flask. On the next day, cells were transfected with a small interfering RNA (siRNA) specific for *CDH3* (50 nm; Hs_CDH3_6, Qiagen, Hilden, Germany; GBML18‐siCDH3) or the scramble siRNA (Qiagen; GBML18‐siCtrl) using lipofectamine 3000 transfection reagent according to manufacturer’s recommendations. Briefly, after the incubation of lipofectamine reagent (lipofectamine/siRNA ratio of 1 : 2) with Opti‐MEM, siRNA diluted in Opti‐MEM was added and incubated for 15 min at RT. This final solution was dropwise added to the cells that were maintained at regular culture conditions. Six hours after transfection, the medium was replaced by fresh complete culture medium and, 48 h after, cells were collected for *CDH3* silencing testing and platted for functional assays.

For lentiviral production, HEK293T cells were seeded at an initial density of 5 × 10^5^ cells per well in 6‐well plate and transfected with 1.2 µg of packaging plasmids (TR30022, Origene, Rockville, MD, USA) and 1 µg of *CDH3*‐specific shRNA [pGFP‐C‐shLenti, Origene; GBML18‐shCDH3 (construct TL314033C), GBML42‐shCDH3‐C (construct TL314033C), or GBML42‐shCDH3‐D (construct TL314033D)] or with a scramble control (TR30021, Origene; GBML18‐shCtrl and GBML42‐shCtrl) using lipofectamine 3000 (as described in Section *CDH3* overexpression in GBM cell line). The lentiviral supernatant was collected three days after transfection and filtered through a 0.45‐µm filter. GBM patient‐derived cells (GBML18 and GBML42), previously platted at the density of 5 × 10^5^ in 25 cm^2^‐flask, were infected with lentivirus in the presence of 8 µg·mL^−1^ polybrene (Sigma‐Aldrich, St. Louis, MO, USA). Successfully infected cells were selected through incubation with puromycin (0.8 µg·mL^−1^ for GBML18 and 1.5 µg·mL^−1^ for GBML42; Santa Cruz Biotechnology).

### 2.5 RNA extraction and cDNA synthesis

RNA of GBM patients’ samples from Hospital de Santa Maria was extracted directly from the tumor piece, while samples from Hospital de Braga were previously converted to powder. RNA from tumor samples, primary cultures and cell lines was extracted using the TRIzol reagent (Invitrogen), following manufacturer’s recommendations. cDNA was synthesized from 1 µg of RNA using the High‐Capacity cDNA Reverse Transcription Kit (Applied Biosystems, Thermo Fisher Scientific, Waltham, MA, USA), following manufacturer’s recommendations.

### 2.6 qRT‐PCR—quantitative reverse transcription‐polymerase chain reactions

*CDH3*, *WNT1*, *WNT5A*, and *TBP* (TATA Box Binding Protein—reference gene) expression levels were determined by qRT‐PCR using TaqMan Fast Advanced Master Mix (1x; Applied Biosystems, Thermo Fisher Scientific). Specific probes were used for *CDH3* (Hs.PT.58.2656432; 1x; IDT, Integrated DNA Technologies, Leuven, Belgium) and *WNT1*, *WNT5A*, and *TBP* (Hs00180529_m1, Hs00998537_m1, and Hs00427620_m1, respectively; 1x; Life Technologies, Thermo Fisher Scientific). The reactions were performed in duplicate using the 7500 Fast Real‐Time PCR software (Applied Biosystems), for 2 min at 50 °C, 20 s at 95 °C, 3 s at 95 °C and 30 s at 60 °C. The last two steps were repeated for 40 cycles. The expression levels of *FZD4*, *PDGFRB*, *ZEB1*, *NANOG*, *VIM*, and *TBP* were assessed using the PowerUp SYBR Green Master Mix (Applied Biosystems). Reactions were performed for 2 min at 50 °C, 2 min at 95 °C, 15 s at 95 °C and 1 min at the respective annealing temperature, and the last two steps repeated for 40 cycles. Primers’ sequences and annealing temperatures are displayed in Table S1. mRNA expression was normalized to *TBP* expression, according to the $2^{‐\Delta C_{t}}$ (Ct: threshold cycle; when presented as relative expression) or $2^{‐\Delta \Delta C_{t}}$ (when presented as fold change) methods [25].

### 2.7 Viability assays

For trypan blue (Gibco, Thermo Fisher Scientific) exclusion assay, cells were plated at an initial concentration of 6 × 10^3^ (U87MG), 1.5 × 10^4^, or 2 × 10^4^ (GBML18, siRNA or shRNA model, respectively), and 6 × 10^3^ (GBML42) per well, in 6‐well plates (each condition in triplicates), and counted 6 days after platting.

For MTS (CellTiter 96® AQueous One Solution Cell Proliferation Assay; Promega, Madison, WI, USA), 4 × 10^3^ GBML18 and 2 × 10^3^ GBML42 cells were platted in 24‐well and 12‐well plates, respectively, and incubated for 6 days. Cultures were incubated with 10% MTS in complete medium for up to 4 h at 37 °C and 5% (v/v) CO_2_. Optical density was measured at 490 nm.

### 2.8 Neurosphere formation assay

Glioblastoma cells were platted at low density (2 × 10^3^ cells per well for U87MG and 1 × 10^3^ cells for GBML18) in low‐attachment 24‐well plates (Corning Inc., Corning, NY, USA), in DMEM‐F12 (Gibco) supplemented with B27 (1%, Invitrogen), EGF (epidermal growth factor, 20 ng·mL^−1^, Invitrogen), and bFGF (basic fibroblast growth factor, 20 ng·mL^−1^, Invitrogen). Fresh medium was added every 2–3 days, and the total number of neurospheres formed was counted at day 10 of incubation for U87MG cells and at day 8 for GBML18.

### 2.9 3D spheroid cell invasion

Spheroids were obtained following manufacturer’s instructions (GravityTRAP™ ULA Plate, InSphero AG, Schlieren, Switzerland). Briefly, 100 or 200 cells/70 µL (U87 and GBML18, respectively) were added to a 96‐well from the GravityTRAP™ ULA Plate and left for 24 h in order to form the cellular spheroid. The medium containing 6–8 spheroids was recovered to a tube. The excess medium was removed, and the spheroids were further embedded in rat tail collagen type I matrix (NaHCO_3_ 0.25 m; NaOH 1 m, Millipore, Burlington, MA, USA). 3D spheroids embedded in collagen type I were plated in an 8‐well coverslip bottom chambers (IBIDI, Planegg, Germany) followed by 1 h of incubation at 37 °C to allow polymerization. After this, complete medium without phenol red was added to the cellular 3D spheroid cultures. The *in vitro* behavior was followed by time‐lapse microscopy (Leica microscope—DMI6000B with Adaptative Focus Control, Wetzlar, Germany) during 18 h. Further, the images taken every 20 min were converted into a time‐lapse movie, and quantitative analysis of the number of isolated cell invasion and protrusions was performed using Leica lasx Software (Leica, Wetzlar, Germany).

### 2.10 Wound healing assay

GBML18 and GBML42 cells were platted at high density (6 × 10^4^ cells in each compartment) in 2‐well inserts (ibidi GmbH, Gräfelfing, Germany) and incubated overnight to allow cells’ adhesion. Inserts were removed on the following day, and migration capacity was registered in photographs taken in an inverted microscope (objective lens magnification 4×; Olympus CKX41 microscope, Olympus, Tokyo, Japan). For each time point, two images/areas were recorded per well. Wound widths (in pixels) were calculated using the bewound software (version 1.7, BESURG, Braga, Portugal), and wound closures were determined in percentages.

### 2.11 Cell cycle

GBML18 and GBML42 cells were platted in 25‐cm^2^ flasks at the initial density of 3 × 10^5^ cells and collected 48 and 32 h after platting, respectively. Cells were fixed in 70% (v/v) ethanol, washed twice with PBS 1x, and incubated with propidium iodide (20 µg·mL^−1^, PI, Invitrogen), RNase (250 µg·mL^−1^, Invitrogen), and Triton X‐100 (0.1%) for 1 h in the dark at 50 °C. Cell acquisition was performed on a BD LSRII flow cytometer (BD Biosciences, Franklin Lakes, NJ, USA) with a 488 nm excitation laser and using the FACS DIVA software (BD Biosciences). At least 30000 gated events were acquired. Data were analyzed using the flowjo Software (v10.8.0; Tree Star, Ashland, OR, USA). Doublet cells were removed plotting FSC‐area vs FSC‐height and double‐checked plotting PI‐area vs PI‐height. The percentage of cells in each phase was obtained applying the Dean‐Jett‐Fox algorithm. Experiments were performed in quadruplicates.

### 2.12 Immunohistochemistry (IHC)

Immunohistochemistry was performed in 3‐μm formalin‐fixed paraffin‐embedded (FFPE) tissue sections as previously described for P‐cadherin [26], and for Ki‐67, NESTIN, SOX2, BCL2, and Cyclin D1 [27] (details about the antibodies and conditions are presented in Table S2). P‐cadherin expression in glioma samples was evaluated and semi‐quantified by an experienced neuropathologist (Dr. Ricardo Taipa). The percentage of P‐cadherin positive cells was defined as: 0–1% and 1–25%. Expression intensity was defined as: negative, mild, and moderate/intense. Normal human skin and breast tissue were used as positive controls. The percentage of stained areas for Ki‐67, NESTIN, SOX2, BCL2, and Cyclin D1 in orthotopic GBM tumors formed upon the injection of U87‐Ctrl or U87‐CDH3 cells in NSG mice were quantified in at least three animals per group (objective lens magnification 20×: median ~ 137 mm^2^; Olympus Upright BX61 microscope) using imagej software (version 1.52a, Bethesda, MD, USA).

### 2.13 Western blot (WB)

Western blot was performed in protein lysates as previously described [28]. Briefly, protein lysates were prepared from cultured cells using a lysis buffer [1% (v/v) Triton X‐100 and 1% (v/v) NP‐40 (Sigma) in deionized water Diagnostics GmbH; 1 : 7 Protease Inhibitors Cocktail (Roche Diagnostics Gmbh, Mannheim, Germany) and 1 : 100 Phosphatase inhibitor (Sigma‐Aldrich, Darmstadt, Germany)]. Cells were washed twice in PBS and were allowed to lyse in 500 μL of the lysis buffer for 10 min, at 4 °C. Lysates were then submitted to vortex, centrifuged at high‐speed and supernatants were collected. Protein concentration was determined using Bio‐Rad protein assay (Bio‐Rad, Richmond, CA, USA). Samples were then separated by 10% SDS/PAGE. Proteins were transferred into nitrocellulose membranes (Amersham Hybond ECL, Amersham Biosciences, Buckinghamshire, UK) at 100 V for 90 min. For immunostaining, membranes were blocked with 5% (w/v) non‐fat dry milk in PBS containing 0.5% (v/v) Tween‐20. Membranes were subsequently incubated with primary antibodies (details in Table S2), followed by six 5‐min washes in PBS/Tween‐20 and incubation with horseradish peroxidase‐conjugated secondary antibodies for 1 h. Membranes were then washed for 30 min in PBS/Tween‐20. Proteins were detected using ECL Chemiluminescence detection kit (Amersham Pharmacia Biotech, Piscataway, NJ, USA) as a substrate. Blots were exposed to an autoradiographic film. Each immunoblot was performed at least three times, and the ones selected for figures are representative experiments.

### 2.14 Mice studies

Mice were kept under standard laboratory conditions with artificial 12 h light/dark cycle (all experiments performed during daylight), controlled ambient temperature (21 ± 1 °C), and a relative humidity of 50–60%, always manipulated in a flow hood chamber, except during intracranial surgeries and ultrasound imaging acquisition. Mice had available *ad libitum* irradiated food and autoclaved water, and were housed in groups of 3–5 per cage to allow social interaction. Cages were also enriched with paper to promote nest building and mice hiding. Sentinel mice health status was used as confirmation of specified pathogen‐free according to FELASA (Federation of European Laboratory Animal Science Associations).

#### *In vivo* subcutaneous GBM xenograft models

To establish the U87 GBM subcutaneous xenograft model in mice, 1 × 10^6^ U87 cells (U87‐Ctrl or U87‐CDH3) were injected in both flanks of male immunodeficient SHrN® hairless NOD.SCID mice (NOD.Cg‐*Prkdc^scid^Hr^hr^*/NCrHsd; Harlan Laboratories; 3 per group) aged 6–8 weeks, randomly distributed between the two groups. Tumors were measured with a caliper, and the tumor volume was determined by assessing the two largest sides [Volume (cm^3^) = (3.14 × L1 × L1 × L2)/6] (L1 = length; L2 = width). All animals were euthanized when the largest side of the tumor of one animal reached 2 cm. After euthanasia, the tumors were collected and fixed by immersion in formalin and subsequently embedded in paraffin for histological analyses.

The subcutaneous patient‐derived xenograft model using GBML18 primary culture (GBML18‐shCtrl or GBML18‐shCDH3) was established by injecting 1.5 × 10^6^ cells in the flank of 4–5 months old male immunodeficient NOD *scid* gamma (NSG) mice—NOD.Cg‐*Prkdc^scid^Il2rg^tm1Wjl^*/SzJ (Charles River Laboratories, Wilmington, MA, USA; 4 GBML18‐shCtrl and 5 GBML18‐shCDH3), randomly distributed between the two groups. Tumors were measured by ultrasound, as detailed below (Section In vivo ultrasound imaging). After euthanasia, the tumors were collected and fixed for histological analyses.

#### *In vivo* ultrasound imaging

The Vevo 3100 ultra‐high‐frequency ultrasound imaging station for small animal (FUJIFILM VisualSonics, Toronto, Canada) was used to assess mice subcutaneous tumor volume at the endpoint. Image acquisitions were performed using the MX550D transducer (40 MHz) and the Mouse (Small) Abdominal settings.

Mice were anesthetized with 3% isoflurane in oxygen, and their flanks were depilated using a depilation cream, and immobilized in the prone position and maintained warm in Vevo’s mouse handling table. Mice physiological status (heart and respiratory rates, and body temperature) was closely monitored during the entire acquisition session. Mice were imaged using the motorized 3D mode. Transducer focal length, as well as the acquisition width and depth, was adjusted to the tumor area. The B‐mode scanning plane was aligned with the 2D cross section showing the tumor at its largest diameter, and the 3D range adjusted to capture the entire tumor of each mouse (step size was automatically set by the Vevo system). Each 3D acquisition was later analyzed in the vevolab software (version 5.5.0, FUJIFILM VisualSonics), using Vevo’s multislice method for tumor volume quantification.

#### *In vivo* intracranial orthotopic GBM xenograft model

The intracranial orthotopic model was obtained by injecting U87‐Ctrl or U87‐CDH3 cells (2 × 10^5^ cells diluted in 5 µL of PBS per animal) stereotactically in the striatum region (+1.8 mm mediolateral, +0.1 mm anteroposterior, −2.5 mm dorsoventral from bregma) of 6‐ to 10‐week‐old male immunodeficient NSG mice (Charles River Laboratories). Three independent experiments were performed with a total number of 18 U87‐Ctrl and 19 U87‐CDH3 animals. Mice were randomly assigned to groups, ensuring no major body weight and age differences between groups. Pre‐surgery procedures included anesthesia with ketamine (75 mg·kg^−1^) and medetomidine hydrochloride (1 mg·kg^−1^) intraperitoneally injected, and the analgesic drug butorphanol (5 mg·kg^−1^) that was subcutaneously injected. Cells were injected using a stereotaxic apparatus (digital 3‐axis; Stoelting, Wood Dale, IL, USA) and a 10 µL Hamilton syringe (point style 4 beveled and 26 s gauge) at the velocity of 1.7 µL·min^−1^. Body weight was measured at least every 2 days. The humane endpoint established was 30% weight loss in relation to the maximum weight reached. When the humane endpoint was achieved, the animals were sacrificed by anesthesia overdose. After perfusion using paraformaldehyde 4%, the brain was collected to be latter embedded in paraffin for histological analyses.

### 2.15 Data mining from publicly available glioma patients’ datasets

*CDH3* expression data obtained by RNA sequencing (Illumina HiSeq 2000 sequencing system) and expression microarrays (Agilent G4502A 244K), and clinical data were downloaded from The Cancer Genome Atlas (TCGA) database (https://portal.gdc.cancer.gov/) [29]. The IDH mutation and 1p19q codeletion status data were extracted from GlioVis (http://gliovis.bioinfo.cnio.es/) [30]. RNA‐Seq expression data contained 5 non‐tumor unmatched controls, 224 WHO grade II and 240 WHO grade III gliomas, and 141 GBMs. *CDH3* overexpression in glioma samples was considered when higher than the maximum expression of nontumor controls in RNA‐Seq data (TCGA FPKM‐UQ value > 28 700). Microarray expression data contained 573 GBMs. The median expression value was used whenever more than one portion per patient was available. Values were pre‐processed and normalized according to ‘level 3’ specifications of TCGA.

The *CDH3* most positively (Spearman *r* > 0.3) and inversely (Spearman *r* < −0.3) correlated genes (microarray data) were used to test for enriched Reactome and KEGG pathways and gene ontologies using Enrichr (https://amp.pharm.mssm.edu/Enrichr/) [31, 32].

### 2.16 Gene set enrichment analysis (GSEA)

Gene set enrichment analysis analysis was performed using the gsea software (www.broad.mit.edu/gsea/) [33] and the microarray expression profile of GBM patients from TCGA (*n* = 573; Agilent G4502A 244K) [29]. A continuous phenotype profile was used; that is, Pearson’s correlation was applied to rank the genes according to *CDH3* expression. Otherwise, default options were used. Gene sets from the molecular signature database C6 collection were used. A gene set was considered significantly enriched when presenting a false discovery rate (FDR) lower than 0.30.

### 2.17 Statistical analysis

To determine statistical differences between groups in the *in vivo* assays, homoscedasticity was tested using the *F* test, and afterward, two‐sided unpaired *t*‐test was applied, except for longitudinal subcutaneous tumor growth, in which a two‐way ANOVA followed by Sidak’s multiple comparisons test was used. For *in vitro* assays, two‐sided paired *t*‐tests were used, except for migration and cell cycle analyses, where a two‐way ANOVA followed by Sidak’s multiple comparisons test was applied. For the OS studies (*in vivo* mice models and GBM patients), *CDH3* effect was determined using the log‐rank (Mantel‐Cox) test (in patients from the Portuguese cohort, the top 30% of samples with lowest expression of *CDH3* were defined as *CDH3*‐low GBMs). The prognostic value of *CDH3* was also evaluated using a Cox multivariable analysis adjusted for confounding factors. All analyses were performed in graphpad prism (GraphPad software, Inc., San Diego, CA, USA, version 8) and ibm spss statistics (Armonk, New York, NY, USA, version 25) software. Meta‐analysis and forest plot were performed in the software Comprehensive Meta‐Analysis (Biostat, Inc., Englewood, NJ, USA, version 3) using hazard ratios and confidence intervals of multivariable analyses (random effect was determined). Each statistical test used is reported in figure legends. Statistically significant differences were considered when *P*‐values were lower than 0.05.

### 2.18 Study approval

Collection of human glioma samples at Hospital de Braga and Hospital de Santa Maria was approved by the respective ethical entity (Subcomissão de Ética para as Ciências da Vida e da Saúde—SECVS 150/2014 and Comissão Nacional de Proteção de Dados—CNPD 7435/2011, respectively) with all patients signing informed consent, according to the Declaration of Helsinki. All animal procedures were approved by the national ethical committee DGAV (Direção Geral de Alimentação e Veterinária, reference no. 017761) and were in accordance with European Union Directive 2010/63/EU.

## 3 Results

### 3.1 *CDH3*/P‐cadherin is overexpressed in a subset of gliomas

**Fig. 1:**
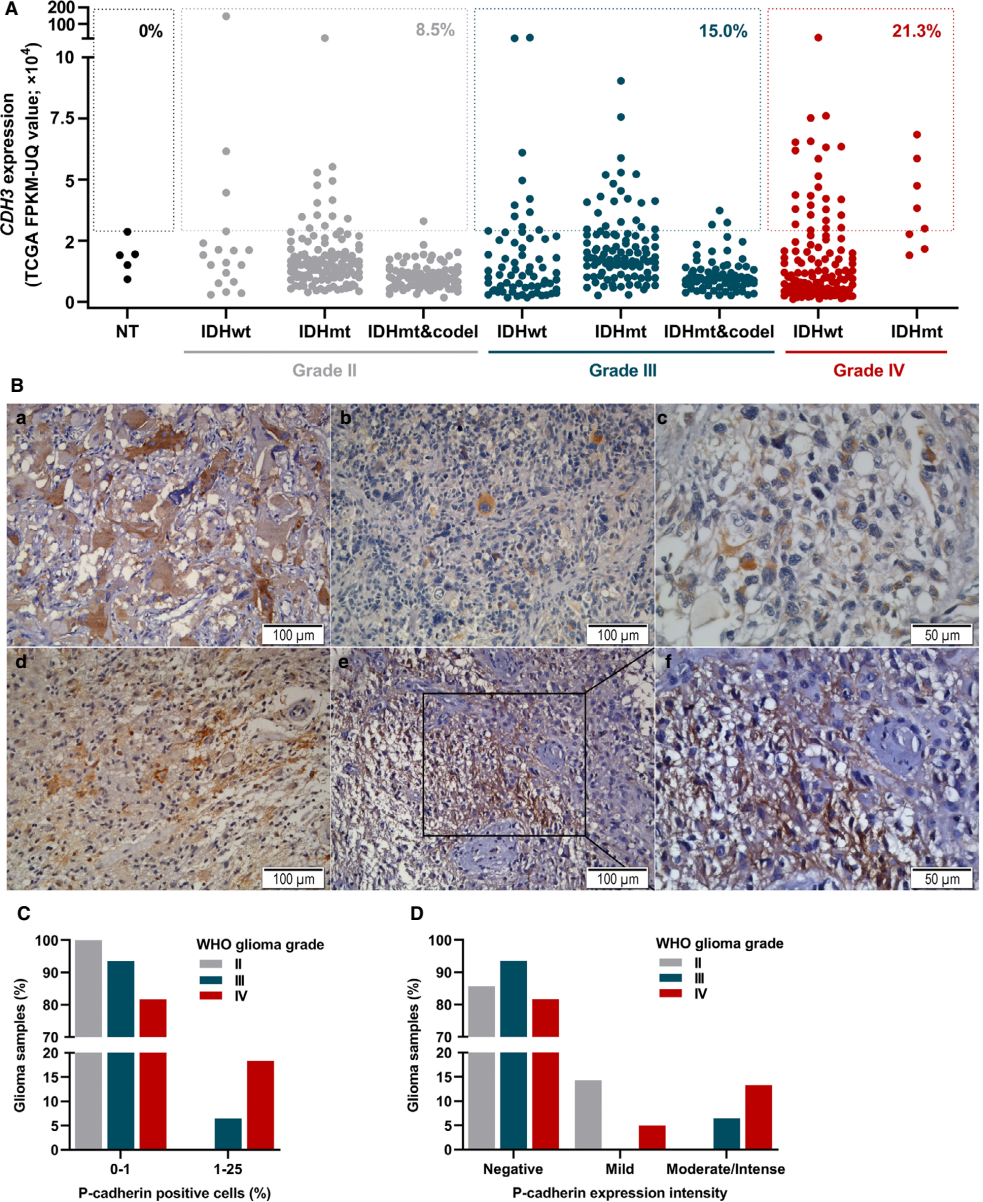
*CDH3*/P‐cadherin is overexpressed in a subset of gliomas. (A) *CDH3* mRNA expression in 5 nontumor controls (NT; black dots) and 605 glioma patients from TCGA (RNA‐Seq data), comprising 224 grade II (gray dots), 240 grade III (blue dots), and 141 grade IV (red dots). Data are stratified according to IDH mutation and 1p/19q codeletion status, and malignancy grade: 18 IDH‐wild‐type, 121 IDH‐mutant 1p19q non‐co‐deleted, and 85 IDH‐mutant 1p19q co‐deleted grade II gliomas; 66 IDH‐wild‐type, 102 IDH‐mutant 1p19q non‐co‐deleted, and 72 IDH‐mutant 1p19q co‐deleted grade III gliomas; 133 IDH‐wild‐type and 8 IDH‐mutant grade IV gliomas. The percentage values indicated for each WHO glioma grade correspond to the proportion of patients overexpressing *CDH3* (expression higher than non‐tumor controls). (B) Representative IHC images of P‐cadherin staining in glioma samples from a cohort of patients from Hospital de Braga (*n* = 98). P‐cadherin staining was mostly cytoplasmic in glioma cells (images a and b) and, more rarely, associated with positivity in fibrillary astrocytic networks (images c‐f; image f is a magnified region of e). Images a, b, c, e, and f show GBM samples, and image d represents an anaplastic oligodendroglioma. Scale bar = 100 µm, except in image c and f in which scale bar = 50 µm. (C–D) Semiquantitative analysis of P‐cadherin IHCs in WHO grades II (*n* = 7), III (*n* = 31), and IV (*n* = 60) gliomas from the Hospital de Braga cohort, showing the percentage of cells expressing P‐cadherin (C), and the staining intensity (D). (TCGA: The Cancer Genome Atlas; FPKM‐UQ: Fragments Per Kilobase of transcript per Million mapped reads upper quartile; codel, chromosomal 1p/19q codeletion; IDHwt, IDH‐wild‐type; IDHmt, IDH‐mutant; NT, non‐tumor; WHO, World Health Organization).

To study the expression pattern of *CDH3* in gliomas, we firstly analyzed its expression levels in patient samples (WHO grades II to IV) deposited in TCGA database with available RNA‐seq data (Fig. 1A). In this analysis, we took into consideration the stratification of patients as per their IDH mutation and 1p/19q codeletion status, according to the 2016 WHO classification of CNS tumors [34]. A wide spectrum of expression of *CDH3* was observed in each glioma subgroup, reflecting the remarkable inter‐individual heterogeneity typical of glioma. Interestingly, particular cases of high expression of *CDH3* were found in each glioma subtype, both in IDH‐wild‐type (22.2%, 16.7%, and 18.8% of IDH‐wild‐type grade II, III, and IV gliomas, respectively) and IDH‐mutant cases (11.6%, 21.6%, and 62.5% of gliomas IDH‐mutant grade II, III, and IV, respectively). In contrast, IDH‐mutant and 1p/19q co‐deleted gliomas, classically classified as oligodendroglioma, presented consistently low levels of *CDH3* expression, with only 1.2% and 4.2% of grade II and III cases overexpressing *CDH3*. Focusing on each WHO malignancy grade, independently of IDH mutation and 1p/19q codeletion status, the frequency of *CDH3* overexpression increased with higher tumor grades (8.5% in grade II, 15.0% in grade III, and 21.3% in grade IV gliomas).

We next evaluated P‐cadherin protein expression by IHC in our cohort of glioma samples from which FFPE‐tissues were available (Hospital de Braga; *n* = 98), including WHO grades II, III and IV gliomas. The major clinicopathological features, including histological subtype and WHO grade (of which GBM constitutes the majority of cases), age and gender distribution, IDH mutation status, treatment exposure and institution are presented in Table 1. We detected P‐cadherin positive samples in all malignancy grades of gliomas (Fig. 1B–D), with a higher frequency within grade IV (GBM) tumors (of note, only 1 out of 7 tested grade II gliomas presented detectable levels of P‐cadherin expression, but only at mild levels and in less than 1% of the cells; Fig. 1C,D). Semiquantitative analyses demonstrated that an increased percentage of P‐cadherin positive cells and higher staining intensity is found in grade IV gliomas (Fig. 1C,D). Representative P‐cadherin IHC images of each WHO glioma grade are shown in Fig. S1.

**Table 1:** Clinical information of patients included in the Portuguese dataset.

Number of patients^a^	115
Diagnosis, *n* (%)	
Diffuse astrocytoma	3 (2.6%)
Anaplastic astrocytoma	3 (2.6%)
Oligodendroglioma	2 (1.7%)
Anaplastic oligodendroglioma	20 (17.4%)
Oligoastrocytoma	1 (0.9%)
Anaplastic oligoastrocytoma	1 (0.9%)
Glioblastoma	85 (73.9%)
WHO Grade, *n* (%)	
II	6 (5.2%)
III	24 (20.9%)
IV	85 (73.9%)
Lesion, *n* (%)	
Primary	108 (93.9%)
Recurrence	7 (6.1%)
Age, mean (range)	59.5 (20–81)
Gender, *n* (%)	
Male	78 (67.8%)
Female	37 (32.2%)
*IDH1*/*IDH2* mutation status^b^, *n* (%)	
wt	42 (36.5%)
mt	15 (13.0%)
Not specified	58 (50.4%)
*MGMT* promoter methylation^c^, *n* (%)	
Methylated	11 (9.3%)
Unmethylated	14 (11.9%)
Not specified	93 (78.8%)
Treatment, *n* (%)	
Chemotherapy	2 (1.7%)
Radiotherapy	16 (13.9%)
Chemoradiotherapy	83 (72.2%)
Not treated	8 (7.0%)
Not specified	6 (5.2%)
Institution, *n* (%)	
Hospital de Braga	72 (62.6%)
Hospital de Santa Maria	43 (37.4%)

^a^ Patients from Hospital de Braga and Hospital de Santa Maria with P‐cadherin protein expression evaluated by IHC (*n* = 71) and *CDH3* mRNA expression evaluated by qRT‐PCR (*n* = 66).

^b^ Determined by restriction fragment length polymorphism (RFLP) or IHC confirmed by Sanger sequencing in dubious cases.

^c^ Determined by pyrosequencing technology [80].

Globally, the increased percentage of *CDH3*/P‐cadherin overexpressing samples in grade IV gliomas suggests this molecule may be relevant for tumor aggressiveness.

### 3.2 P‐cadherin is associated with GBM *in vitro* aggressiveness in an overexpression model

Considering the heterogeneity observed in patients, we firstly studied the expression of *CDH3* in a total of 12 GBM *in vitro* cell models, including six GBM cell lines and six patient‐derived cultures (Fig. S2). We identified GBM cells with high (SNB19, GBML18, GBML24, and GBML45), intermediate (A172, GBML26, GBML42, and GBML95), and low/undetectable *CDH3* expression (U87MG, U373MG, U251MG, and LN229), nicely recapitulating the heterogeneity observed in patients.

**Fig. 2:**
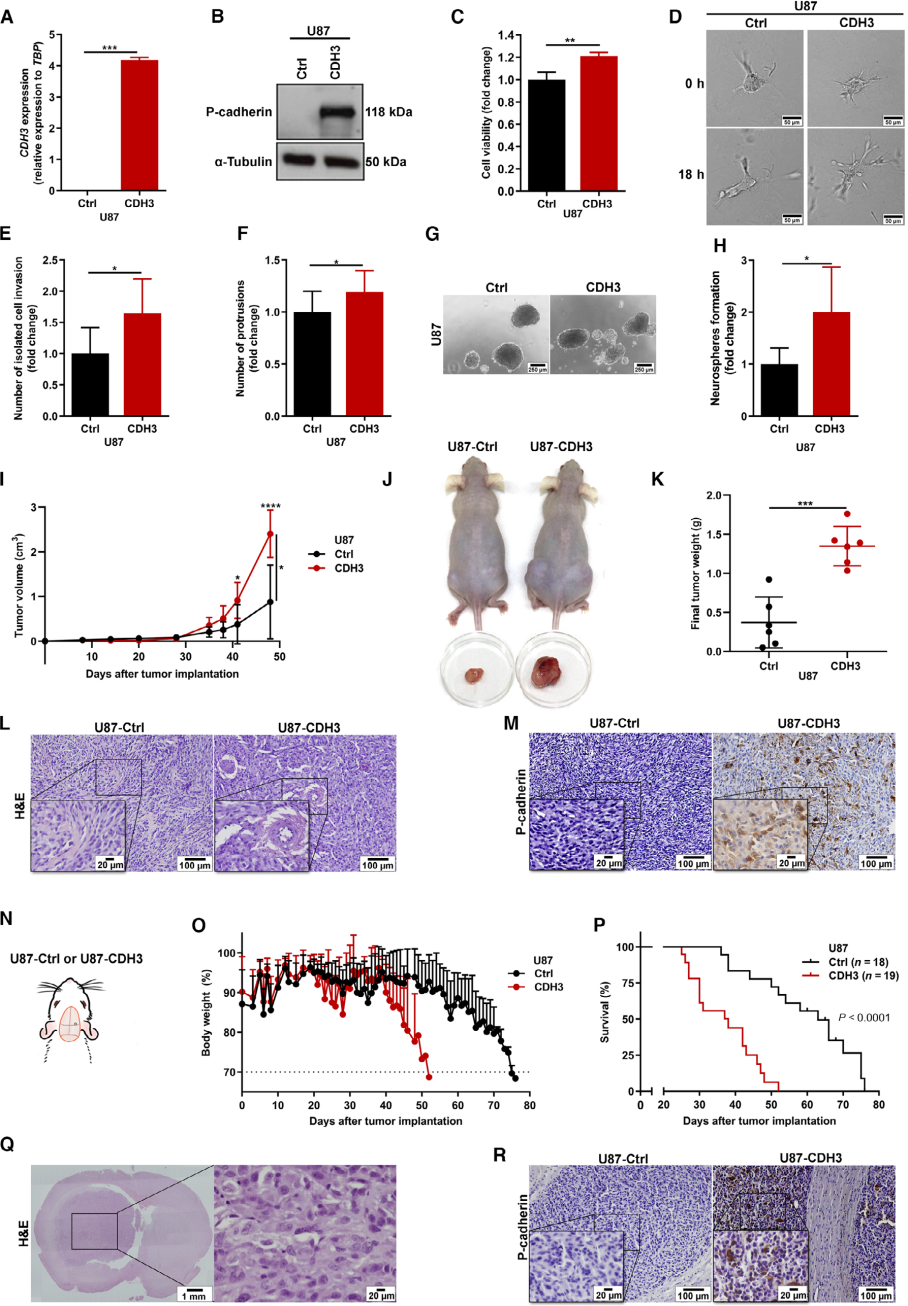
P‐cadherin displays oncogenic functions by regulating key hallmarks of malignancy in GBM *in vitro* and *in vivo*. (A–B) Successful *CDH3* overexpression in the GBM cell line U87MG, as assessed at the mRNA levels by qRT‐PCR (*n* = 3) (A) and at protein levels by western blot (*n* = 3, representative experiment is displayed) (B). (C) Effect of P‐cadherin in cell viability assessed by trypan blue in U87 cells (*n* = 4). (D–F) Three‐dimensional invasion assays of P‐cadherin‐high and P‐cadherin‐low GBM cells (*n* = 5; representative images are shown for both conditions (D); scale bar = 50 µm). Quantification of isolated cell invasion (E) and protrusions (F) in GBM cells with high and low P‐cadherin expression. (G–H) Influence of P‐cadherin in the stemness capacity of GBM cells as assessed by the neurosphere formation assay (*n* = 5; representative images of all conditions are shown (G); scale bar = 250 µm). (H) Quantification of the number of neurospheres formed in GBM cells with high and low levels of P‐cadherin. (I) Effect of P‐cadherin on tumor growth kinetics of *in vivo* subcutaneous GBM models (bilateral injections per mice, *n* = 3 hairless NOD.SCID mice per group). (J) *In vivo* and *ex vivo* pictures at the experimental endpoint (day = 49) of U87 subcutaneous tumors with high or low P‐cadherin expression levels (bilateral injections per mice, *n* = 3 per group). (K) Final tumor weight of subcutaneous tumors formed from U87‐Ctrl and U87‐CDH3 cells (bilateral injections per mice, *n* = 3 per group). (L) H&E staining of subcutaneous tumors (representative images are displayed; scale bars = 100 µm and 20 µm, as indicated). (M) P‐cadherin protein expression in the subcutaneous U87‐Ctrl and U87‐CDH3 tumors (representative images are displayed; scale bars = 100 µm and 20 µm, as indicated). (N) Intracranial surgery scheme for orthotopic models (*n* = 18 for U87‐Ctrl, and *n* = 19 U87‐CDH3). (O) Total body weight variation after intracranial injections of *CDH3*‐manipulated U87 cells in NSG mice (*n* = 18 for U87‐Ctrl, and *n* = 19 for U87‐CDH3). (P) Overall survival of mice intracranially implanted with *CDH3*‐overexpressing or control‐U87 GBM cells (*n* = 18 for U87‐Ctrl, and *n* = 19 for U87‐CDH3). (Q) H&E staining of brain sections to confirm tumor formation (representative images are displayed; scale bars = 1 mm and 20 µm, as indicated). (R) Long‐term P‐cadherin expression in GBM tumors formed from U87‐Ctrl and U87‐CDH3 cells’ mice orthotopic injection (representative images are displayed; scale bars = 100 and 20 µm, as indicated). [H&E: hematoxylin and eosin staining; panels A, C, E, F,H two‐sided paired *t*‐test, data is presented as mean ± SEM; panel K: two‐sided unpaired *t*‐test, data are presented as mean ± SD; panel I: two‐way ANOVA followed by post‐hoc Sidak’s test, data are presented as mean ± SD; panel O: data are presented as mean ± SD; panel P: log‐rank test; *P < 0.05; **P < 0.01; ***P < 0.001; ****P < 0.0001].

To pinpoint the impact of P‐cadherin in GBM, we firstly performed *in vitro* functional assays using an overexpression approach, in which *CDH3*/P‐cadherin was overexpressed in U87 cells (a commercially available GBM cell line with very low or undetectable levels of *CDH3*/P‐cadherin by qRT‐PCR and western blot). Success of transfection and overexpression was confirmed at the mRNA level by qRT‐PCR (Fig. 2A) and at the protein level by western blot (Fig. 2B). P‐cadherin expression was associated with increased GBM cell viability, as assessed by trypan blue exclusion assay (Fig. 2C). Considering that GBMs are known for their remarkable invasiveness capacity [35], and since P‐cadherin has been shown to impact cell invasion in different tumors (e.g., melanoma [20, 21] and breast cancer [18]), we also evaluated how P‐cadherin affects this cancer hallmark feature by three‐dimensional invasion assays (Fig. 2D). P‐cadherin was associated with increased cell invasion capacity in GBM cells, as measured by isolated cell invasion events (Fig. 2E) and the total number of protrusions (Fig. 2F). P‐cadherin‐overexpressing GBM cells also formed more neurospheres than control cells (Fig. 2G,H). Together, these results suggest that P‐cadherin regulates several critical hallmarks of cancer in GBM *in vitro*, acting mostly in an oncogenic manner.

### 3.3 P‐cadherin promotes tumor growth *in vivo* and associates with shorter survival in GBM xenograft mouse models

A subcutaneous GBM xenograft model was established in immunodeficient hairless NOD.SCID mice injected with U87 cells previously manipulated for *CDH3* expression. Tumors derived from U87‐CDH3 cells presented a significantly higher growth rate than U87‐control tumors (Fig. 2I), resulting in tumors with higher volumes (Fig. 2J) and weights (Fig. 2K) at the humane endpoint of the experiment. Tumor histology (Fig. 2L) and long‐term P‐cadherin protein expression (Fig. 2M) was confirmed.

To better mimic the complex GBM microenvironment, we also established an intracranial orthotopic model in immunodeficient NSG mice injecting CDH3‐low and CDH3‐high U87 GBM cells (Fig. 2N). Mice body weight was registered throughout the experiment, showing that mice injected with U87‐CDH3 cells displayed significant weight loss earlier and more drastically than animals injected with U87‐Ctrl cells (Fig. 2O). More importantly, mice with tumors overexpressing P‐cadherin exhibited significantly shorter survival than mice injected with control GBM cells (*P* < 0.0001; Fig. 2P), suggesting P‐cadherin may be prognostically valuable in GBM. Tumor formation was confirmed through hematoxylin and eosin (H&E) staining of the brains (Fig. 2Q), and P‐cadherin long‐term *in vivo* expression was confirmed in mice tumors by IHC, being exclusively detected in U87‐CDH3 tumors (Fig. 2R). Additionally, we evaluated the expression of some cancer‐related proteins in *ex vivo* tumors from both groups, including proliferation (Ki‐67 and Cyclin D1), anti‐apoptotic (BCL2), and stemness (NESTIN and SOX2) markers, collected at distinct time points according to animal survival times (Fig. S3A,B). Interestingly, despite the prominent effect in the survival of mice, there were no significant differences regarding the levels of expression of these proteins between both groups of tumors at the humane endpoint. Of note, intracranial tumors revealed to be highly heterogeneous both histologically and molecularly, with various levels of positive staining for each marker, regardless of P‐cadherin status (Fig. S3B).

### 3.4 *CDH3* knockdown impairs distinct cancer hallmarks in GBM cells

**Fig. 3:**
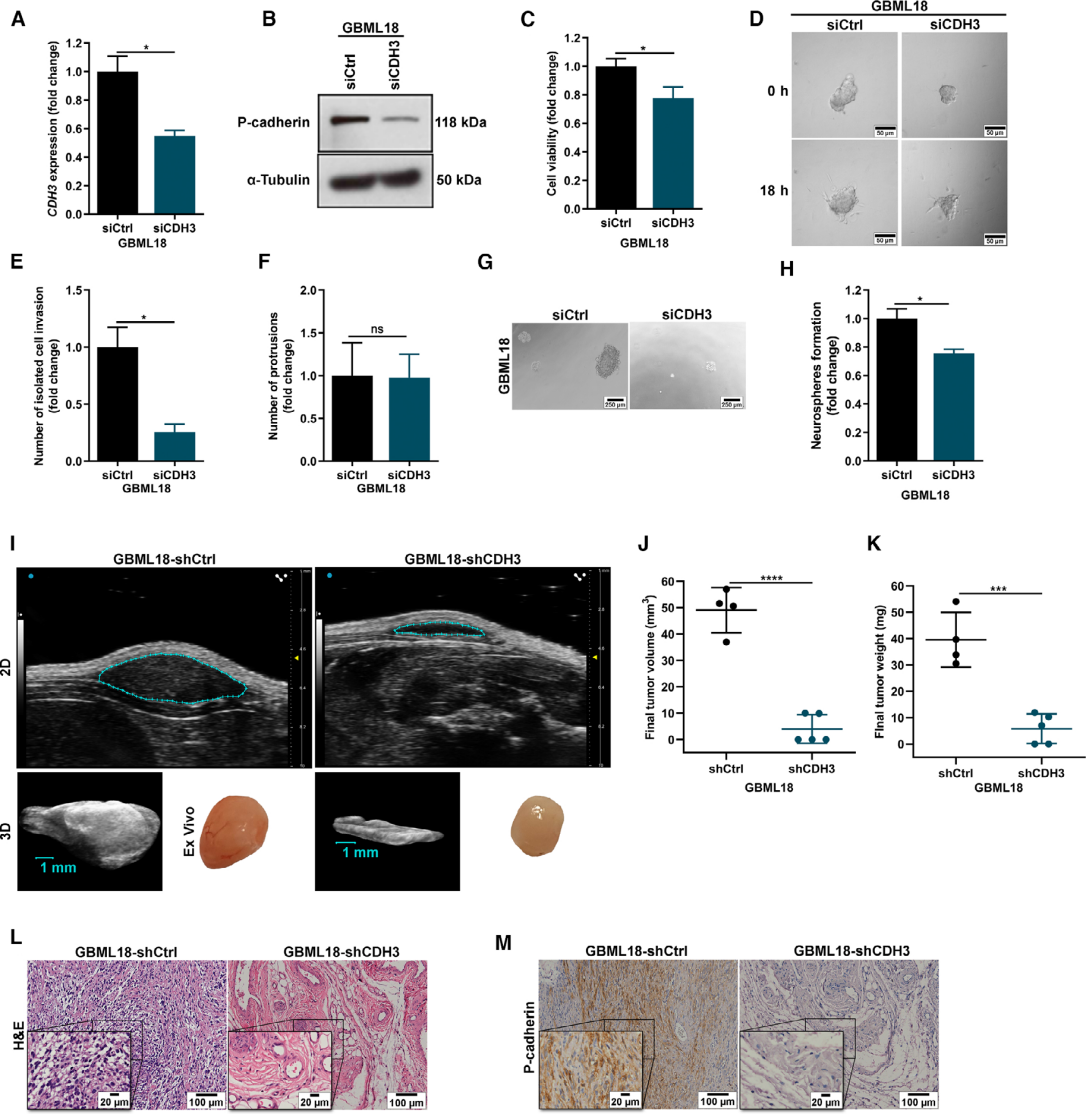
P‐cadherin knockdown impairs distinct cancer hallmarks in GBM cells and diminishes tumor growth *in vivo*. (A–B) Success of transient transfections with siRNA in GBM patient‐derived culture GBML18 assessed by RNA (*n* = 4) (A) and protein levels (*n* = 3, representative experiment is displayed) (B). (C) Functional effect of P‐cadherin in cell viability (trypan blue exclusion assay, *n* = 5). (D–F) Three‐dimensional invasion assay of GBML18‐siCtrl and GBML18‐siCDH3 [*n* = 3; representative images are shown for each experimental condition (D); scale bar = 50 µm]. Isolated cell invasion (E) and protrusions were quantified (F). (G–H) Neurospheres formation capacity (*n* = 5; representative images in (G); scale bar = 250 µm; and quantification of the number of neurospheres formed in each condition (H). (I) Representative ultrasound images obtained at the endpoint of the experiment for subcutaneous tumors formed from the injection of GBML18 cells stably silencing *CDH3* (GBML18‐shCtrl vs GBML18‐shCDH3) in NSG mice (*n* = 4 and 5, respectively), at the largest two‐dimensional cross‐sectional plan and after three‐dimensional reconstruction (scale bar = 1 mm). Respective *ex vivo* tumors are shown. (J) Final tumor volume determined by ultrasound after three‐dimensional reconstruction and measurements (*n* = 4 for GBML18‐shCtrl and *n* = 5 for GBML18‐shCDH3). (K) Final tumor weight of the P‐cadherin‐high and P‐cadherin‐low subcutaneous tumors determined at the endpoint (*n* = 4 for GBML18‐shCtrl and *n* = 5 for GBML18‐shCDH3). (L) Histology of the tumors formed assessed by H&E (representative images are displayed; scale bars = 100 µm and 20 µm, as indicated). (M) IHC showing P‐cadherin expression in GBML18‐shCtrl tumors and silencing in GBML18‐shCDH3 group (representative images are displayed; scale bars = 100 µm and 20 µm, as indicated). [H&E: hematoxylin and eosin staining; panels A, C, E, F, H: two‐sided paired *t*‐test, data are presented as mean ± SEM; panels J, K: two‐sided unpaired *t*‐test, data are presented as mean ± SD; ns: nonsignificant; *P < 0.05; ***P < 0.001; ****P < 0.0001].

In addition to the overexpression approach, the impact of P‐cadherin in GBM cells was also assessed using gene silencing approaches. Firstly, *CDH3* was silenced by siRNA in GBML18 cells (a GBM patient‐derived culture with high levels of P‐cadherin expression). Efficacy of transient transfections was determined at the RNA (Fig. 3A) and protein (Fig. 3B) levels. Fitting well with the findings in the overexpression model using U87MG cells, the silencing of P‐cadherin in GBML18 patient‐derived cultured with siRNA resulted in decreased cell viability (Fig. 3C), decreased invasion (Fig. 3D–F), and decreased capacity to form neurospheres (Fig. 3G,H).

Considering the observed effects of transient silencing of P‐cadherin with siRNA *in vitro*, we next established a stable knockdown model using a shRNA targeting *CDH3*. After confirming the long‐term knockdown of *CDH3*/P‐cadherin (Fig. S4A,B), this shRNA‐based GBML18 model was validated *in vitro* for key cancer hallmarks, globally recapitulating the association of P‐cadherin silencing with compromised GBM cell viability (Fig. S4C,D) and migration capacity (Fig. S4E,F). Furthermore, we confirmed the functional effects of shRNA‐mediated *CDH3* silencing in an additional GBM patient‐derived culture (GBML42). The stable silencing of *CDH3* was confirmed (Fig. S5A), and *CDH3* functional effects were consistently validated, endorsing its association with increased cell viability (Fig. S5B,C), and migration (Fig. S5D,E).

### 3.5 The stable silencing of *CDH3* disrupts GBM tumor growth *in vivo*

To understand the effects of *CDH3* stable silencing *in vivo*, we then established subcutaneous xenograft tumors by injecting GBML18 cells (GBML18‐shCtrl or GBML18‐shCDH3) in the flank of NSG mice. Concordantly to the results obtained with the overexpression model (Fig. 2), mice bearing *CDH3*‐low cells formed much smaller tumors than the ones injected with *CDH3*‐high cells, as observed by ultrasound imaging, both at two‐dimensional analyses and after three‐dimensional tumor reconstructions (Fig. 3I). The collected tumors were consistently smaller in GBML18‐shCDH3 group comparing to GBML18‐shCtrl animals, as proven by the reduced final tumor volume (Fig. 3J), and final tumor weight (Fig. 3K). Tumor histology (Fig. 3L) and P‐cadherin long‐term silencing (Fig. 3M) were confirmed by H&E staining and IHC, respectively.

### 3.6 *CDH3* influences cell cycle progression in GBM cells

Considering the consistent data in distinct models associating P‐cadherin with cell viability and GBM tumor growth *in vivo*, the influence of P‐cadherin silencing on cell cycle was also evaluated in our two GBM primary cultures with stable *CDH3* knockdown (Fig. S6). Curiously, the silencing of *CDH3* significantly impacted cell cycle in the two primary cultures, despite cell‐specific differences. Specifically, the silencing of *CDH3* in GBML18 cells (Fig. S6A) led to a diminished G0/G1 phase and G2/M enrichment (a hallmark of cells undergoing mitotic cell death and with decreased mitosis/proliferation [36, 37]), while in GBML42 (Fig. S6B), *CDH3* knockdown led to an increment of S phase associated with decreased G2/M phase, suggesting an arrest in the DNA replication phase [38].

### 3.7 *CDH3*‐associated gene signatures are enriched for common GBM‐related pathways

**Fig. 4:**
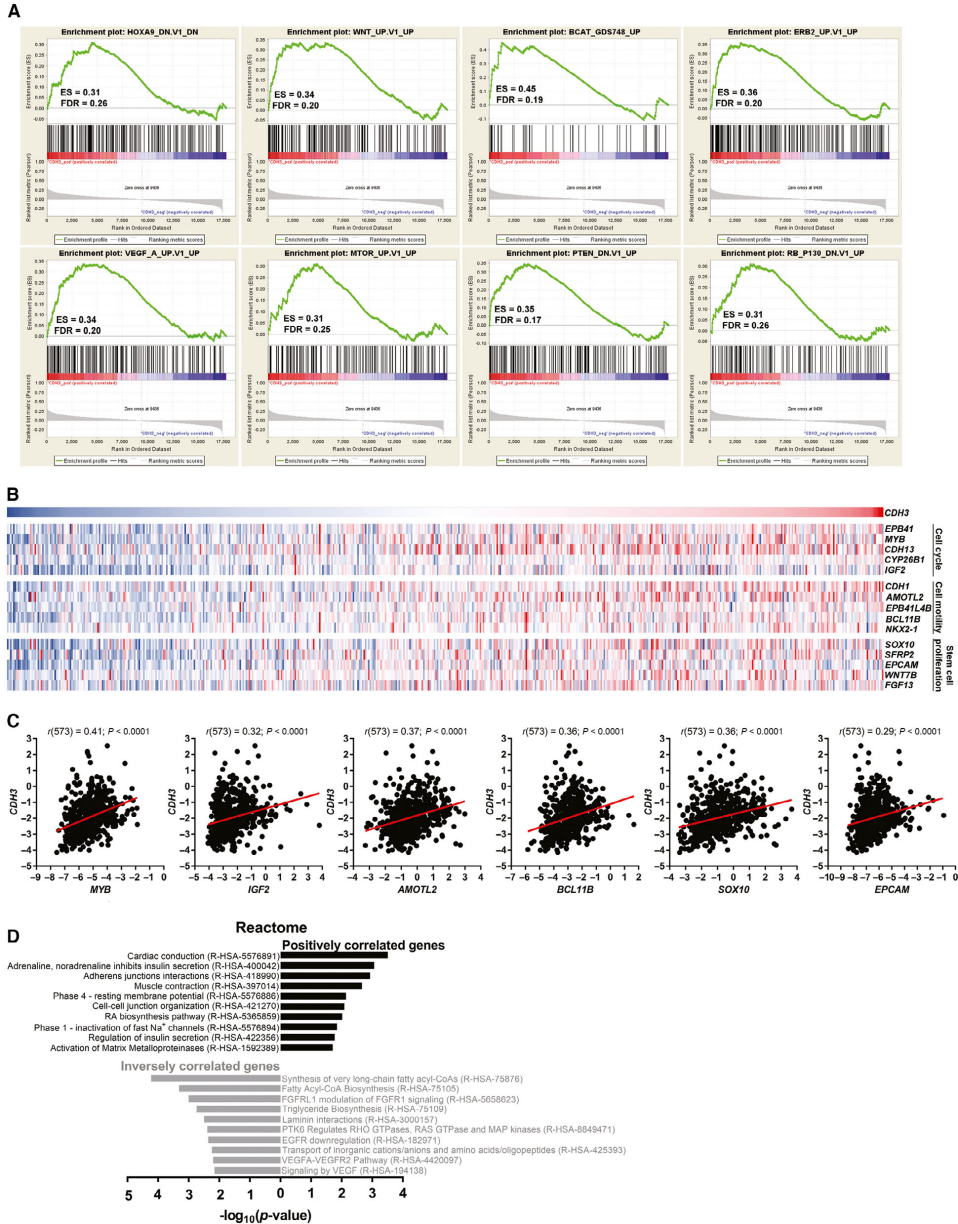
*CDH3*‐positively correlated genes in GBM patients are enriched for genes involved in common cancer‐related pathways. (A) GSEA analyses demonstrating that *CDH3*‐positively correlated genes are enriched for genes downregulated upon the knockdown of *HOXA9*, and for genes upregulated upon the activation of the WNT‐β‐catenin signaling and RTK/PI3K/mTOR signaling axis, or the silencing of *PTEN* and *RB*. (B) Heatmaps of expression levels of *CDH3* and the 5 most positively correlated genes of three distinct GO gene sets (cell cycle—GO:0007049; cell motility—GO:0048870; stem cell proliferation—GO:0072089) in 573 GBM TCGA patients. Darkest blue represents the lowest expression values and darkest red represents the higher expression values of each row. (C) Correlations plots between *CDH3* expression and *MYB*, *IGF2*, *AMOTL2*, *BCL11B*, *SOX10*, and *EPCAM* in TCGA GBM patients (Spearman’s correlations *r* and *P* values are shown). (D) Reactome pathways among the most positively (Spearman *r* > 0.3) and inversely (Spearman *r* < −0.3) *CDH3*‐correlated genes in 573 GBM patients from TCGA (DN, downregulated; ES, enrichment score; FDR, false discovery rate; GO, gene ontology; GSEA, gene set enrichment analyses; TCGA, The Cancer Genome Atlas; UP, upregulated).

To gain insights into putative cellular and molecular mechanisms responsible for the oncogenic effects of P‐cadherin observed *in vitro* and *in vivo*, we performed GSEA in the large cohort of GBM patients from TCGA (*n* = 573). Using *CDH3* expression as continuous label, we found that *CDH3*‐positively correlated genes were enriched for signatures of genes upregulated upon the activation of the WNT‐β‐catenin signaling, RTK/PI3K/mTOR signaling axis (through *ERBB2*, *VEGF* and *mTOR*), and upon inactivation of the tumor‐suppressor genes *PTEN* and *RB*; in addition, *CDH3*‐positively correlated genes were enriched for signatures of genes downregulated upon HOXA9 silencing (Fig. 4A).

To validate our *in vitro* results suggesting that P‐cadherin influences cell viability, cell cycle, invasion, and stemness capacity, we investigated the expression levels of specific genes belonging to three Gene Ontology (GO) gene sets related to those cancer hallmarks. Interestingly, in GBM patients, *CDH3* was co‐expressed with several genes related to cell cycle, cell motility, and stem cells proliferation (heatmaps representing *CDH3*’s top 5 most co‐expressed genes belonging to each gene set, Fig. 4B; and representative individual examples of correlation graphs, Fig. 4C). To further complement these data, we searched for gene ontologies and pathways associated with the *CDH3* most positively and negatively correlated genes (Fig. 4D and Fig. S7). In line with our previous data, we observed that *CDH3*‐positively correlated genes are enriched for Reactome classes such as cell–cell junction organization, and activation of matrix metalloproteinases (Fig. 4D), as well as with other cancer‐related pathways revealed by KEGG analyses, such as cGMP‐PKG and Wnt signaling pathways (Fig. S7A). Finally, the expression of particular key players involved in the signaling pathways and/or biological processes identified in the transcriptomic data from this large dataset of GBM patients was also tested in our genetically manipulated GBM patient‐derived cultures (Fig. S7E). Despite the differences observed between the two knockdown models, suggesting that some of these expression patterns might be cell‐dependent, the overall association between *CDH3* and markers of these pathways was validated (e.g., *CDH3* silencing led to a decreased expression of WNT/β‐catenin genes, such as *WNT1* and *WNT5A*), establishing good parallels between our *in vitro* data and that from patients.

Globally, these genome‐wide analyses suggest that *CDH3*/P‐cadherin might be associated with pro‐tumor gene expression signatures that are known to be critical in GBM signaling.

### 3.8 *CDH3* is predictive of shorter survival in primary GBM patients

**Fig. 5:**
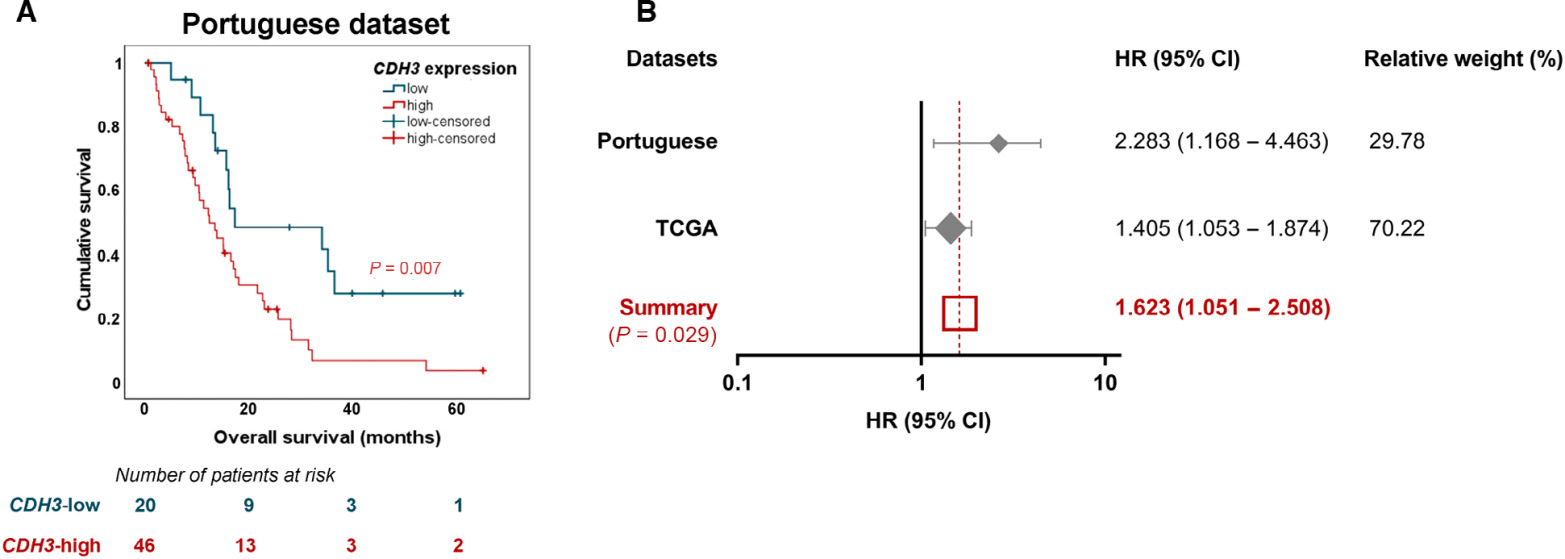
High *CDH3* expression is associated with shorter overall survival in GBM patients. (A) Kaplan‐Meier survival curve of our Portuguese cohort (*n* = 66) regarding low (*n* = 20) and high (*n* = 46) *CDH3* mRNA expression levels in GBM patients, as evaluated by qRT‐PCR. The cohort includes 33 IDH wt (50%), and 33 GBM samples with IDH status not specified (50%); 9 *MGMT* promoter methylated (13.6%), 9 *MGMT* promoter unmethylated (13.6%), and 48 GBM samples not evaluated for *MGMT* promoter methylation (72.7%). (B) Meta‐analysis of the association of *CDH3* and overall survival in GBM patients from the Portuguese and TCGA datasets. The analysis was performed with the hazard rations obtained with Cox multivariable analyses. Each diamond represents one dataset and its size correlates to the relative weight of the dataset in the analysis. The extending lines indicate the 95% confidence intervals. The red square represents the overall effect (TCGA, The Cancer Genome Atlas; HR, hazard ratio; CI, confidence interval; log‐rank test was applied in panel A).

Taking into consideration our data implicating P‐cadherin with GBM aggressiveness *in vitro*, and to be associated with poor prognosis (shorter OS) in a GBM xenograft model, we questioned if this association was also present in human patients. Interestingly, P‐cadherin was already described to associate with poor patient prognosis in other types of cancer [19, 39, 40]. To test our hypothesis, we evaluated *CDH3* mRNA expression levels by qRT‐PCR in primary GBM samples collected at Hospital de Braga and Hospital de Santa Maria (*n* = 66; clinical information in Table 1). The median OS of primary GBM patients in our cohort was 13.7 months, very similar to that described more recently in the literature [3]. We found that GBM patients with high *CDH3* expression present significantly shorter OS than those with low expression levels (Fig. 5A; *P* = 0.007). Importantly, considering the putative effects of age and gender in a multivariable survival analysis, *CDH3* maintained its independent prognostic value (Table 2; *P* = 0.016). This association of *CDH3* with poor prognosis was further validated in the large TCGA dataset using a multivariable Cox model (Table 3; *P* = 0.021), adjusted for the potential confounding effects of *IDH1* mutation status, age at diagnosis, Karnofsky performance status (KPS), gender, treatment with chemo‐ or radiotherapy, and additional treatments. Importantly, to integrate data from the independent cohorts, a meta‐analysis was performed, validating that *CDH3* is significantly associated with shorter OS (Fig. 5B, HR = 1.623, *P* = 0.029). Globally, our data indicate that *CDH3* associates with shorter OS of GBM patients, suggesting its potential as a novel biomarker predictive of poor prognosis in these patients.

**Table 2:** Cox multivariable survival analysis in GBM patients from Portuguese dataset (qRT‐PCR data; *n* = 66). Bold‐faced values indicate significant *P*‐values (CI, Confidence Interval; HR, Hazard Ratio).

	*P*‐value	HR	95% CI
*CDH3* expression^a^, ^b^	**0.016**	2.283	1.168–4.463
Age at diagnosis	**0.047**	1.035	1.001–1.070
Gender^a^, ^c^	0.167	0.619	0.314–1.222

^a^ *CDH3* expression and gender were used as categorical variables.

^b^ *CDH3*‐low (*n* = 20) vs. *CDH3*‐high expression (*n* = 46).

^c^ Male (*n* = 49) vs. Female (*n* = 17).

**Table 3:** Cox multivariable survival analysis in GBM patients from TCGA (microarray data; *n* = 275). Bold‐faced values indicate significant *P*‐values (CI, confidence interval; HR, hazard ratio).

	*P*‐value	HR	95% CI
*CDH3* expression^a^, ^b^	**0.021**	1.405	1.053–1.874
*IDH1* mutation status^a^, ^c^	**0.031**	0.518	0.285–0.941
Age at diagnosis	**0.001**	1.021	1.009–1.034
KPS^a^, ^d^			
0–50	**0.001**	–	–
60–70	**0.004**	0.339	0.162–0.711
80–100	**< 0.001**	0.250	0.122–0.512
Gender^a^, ^e^	0.141	0.793	0.583–1.080
Chemo‐ or radiotherapy^a^, ^f^	**< 0.001**	0.211	0.122–0.367
Additional chemo‐ or radiotherapy^a^, ^g^	0.072	0.762	0.566–1.024

^a^ *CDH3* expression, *IDH1* mutation status, KPS, gender, chemo‐ or radiotherapy, and additional chemo‐ or radiotherapy were used as categorical variables.

^b^ *CDH3*‐low (*n* = 129) vs. *CDH3*‐high expression (*n* = 146), defined based on the median value of expression.

^c^ *IDH1*‐wild‐type (*n* = 252) vs. *IDH1*‐mutant (*n* = 23).

^d^ KPS 0–50 (*n* = 13); 60–70 (*n* = 63); 80–100 (*n* = 199).

^e^ Male (*n* = 172) vs. Female (*n* = 103).

^f^ No treatment (*n* = 18) vs. Treatment (*n* = 257).

^g^ No additional treatment (*n* = 152) vs. Additional treatment (*n* = 123).

## 4 Discussion

Distinct cadherins are known to play a role in gliomas [41, 42, 43, 44, 45, 46]. However, the impact of P‐cadherin, which has been implicated in several types of cancer [20, 47, 48, 49, 50, 51], was still unknown in glioma. While cadherin‐6 [52], cadherin‐11 [52], T‐cadherin [53], R‐cadherin [52], and N‐cadherin [54] are widely expressed in the brain of distinct species, P‐cadherin has been considered largely undetected in various brain regions in mice and humans [10, 13, 55, 56]. Thus, the aberrant expression observed in some gliomas, particularly grade IV gliomas (Fig. 1) may have functional consequences impacting glioma pathophysiology. Other studies reported that P‐cadherin is associated with high histological grades in invasive ductal breast carcinoma [51], lung invasive adenocarcinoma [39], and in colorectal tumors [48]. Concordantly with the idea that P‐cadherin associates with more aggressive cancers, our study established P‐cadherin as a novel oncogenic player in GBM, affecting cell viability, cell cycle, cell invasion, migration, and neurosphere formation (Figs 2 and 3; Figs S4–S6). These data are concordant with the described effect of P‐cadherin in breast cancer, in which it is associated with increased invasiveness and stemness capacity [16, 17, 51]. Contrarily, P‐cadherin associates with decreased cell invasion in melanoma [20]. Considering the relevant impact of P‐cadherin in stemness in other types of cancer, future studies to specifically address how P‐cadherin influences self‐renewal capacity of glioma stem cells are warranted. Despite the *in vitro* results and the prominent effect in shortening mice OS (Fig. 2), we did not find significant differences between control‐ and P‐cadherin‐overexpressing U87 orthotopic tumors regarding the expression of proteins commonly altered in GBM, including stemness markers (Fig. S3). This result may be partly explained by the fact that all tumors (formed from U87‐Ctrl and U87‐CDH3 cells) were analyzed at different time points based on the OS of each animal, a stage at which they have reached similar levels of aggressiveness. Future studies should focus on studying the effect of P‐cadherin in GBM in mice survival using long‐term knockdown approaches.

Cadherins are not considered to have only structural functions. Indeed, different intracellular mediators were reported to interact with P‐cadherin (reviewed in [15]). Consistent with this idea, we found that some of the P‐cadherin oncogenic effects observed in GBM may result from alterations in critical intracellular signaling pathways. Interestingly, our GSEA analyses in GBM patients (Fig. 4) demonstrated associations between *CDH3*‐correlated genes and β‐catenin‐dependent WNT pathway, described to be associated with GBM aggressiveness [57]. This putative link between P‐cadherin and β‐catenin was also previously reported in mammary basal epithelial cells [58], and in colon cancer cells, where the inhibition of P‐cadherin leads to the downregulation of β‐catenin [59]. Furthermore, WNT genes, such as *WNT5A* [60] and *WNT6* [27], were also already described to impact GBM aggressiveness, some of which presented differential expression due to *CDH3* status in our GBM models (Fig. S7E). Interestingly, the associations observed between *CDH3*‐correlated genes, *VEGF*, *ERBB2*, and *mTOR* suggest its involvement in oncogenic pathways, such as the RTK/PI3K/mTOR signaling axis [61]. Moreover, the oncogenic value of *CDH3* is reinforced by the upregulation of *CDH3*‐positively correlated genes upon knockdown of *RB* and *PTEN* tumor‐suppressor genes, and by the association with *HOXA9*, a transcription factor with critical oncogenic functions in GBM [22, 62, 63]. Data from clinical specimens also suggested that *CDH3* correlates with genes involved in key biological processes, such as *MYB*, described to be activated in some GBM cell lines [64], and dependent on PI3K [65], further validating our GSEA data. *IGF2*‐high expressing GBMs are frequently associated with poor survival [66], and IGF2 can interact with IGF‐1R, activating it and leading to a proliferative phenotype through AKT [67]. Interestingly, P‐cadherin on its own is described to activate invasion and metastasis capacities in ovarian cancer by interacting with IGF‐1R [68]. In addition to these specific genes, we identified a strong positive correlation between *CDH3* and genes involved in cell migration (*AMOTL2* [69] and *BCL11B* [70]] and stemness capacity [*SOX10* [71] and *EPCAM* [72]) in GBM patients, further supporting our *in vitro* data. While these findings result from association and correlation analyses from patient samples, and thus do not establish direct causative relationships, they may shed light into the gene signatures and signaling pathways by which P‐cadherin mediates its oncogenic functions in glioma, which could be further explored in future studies.

Despite the advances that resulted in the identification of clinically relevant molecules in GBM, the heterogeneity of these tumors still requires the discovery of novel players to improve the accuracy of diagnosis and prognosis [73]. The prognostic value of *CDH3*, predictive of shorter OS in independent cohorts of GBM patients (Fig. 5 and Tables 2 and 3), is concordant with the results obtained *in vivo*, and fits well with previous reports in breast cancer [19, 40] and lung cancer [39]. The prognostic value of other classical cadherins, such as E‐ and N‐cadherin, has been explored in gliomas, but somewhat contradicting findings have been reported, with some authors, suggesting that these cadherins are not suitable as prognostic molecules [74], while others report low levels of *CDH2* (coding N‐cadherin protein) as being predictive of longer survival and better response to temozolomide [45], the most used chemotherapeutic agent in the treatment of GBM.

Our findings implicating P‐cadherin as a tumor promoting molecule, and the prognostic value of *CDH3* independent of other known prognostic indicators, suggest this cell–cell adhesion molecule may be an attractive new therapeutic target for P‐cadherin‐positive tumors. Thus, in future, it will be critical to test the therapeutic value of specific inhibitors of P‐cadherin in GBM, some of which have been already developed [75, 76, 77, 78, 79] and completed phase 1 clinical trials [e.g., an immunoconjugate PCA062 (NCT02375958) and an anti‐P‐cadherin/anti‐CD3‐bispecific molecule PF‐06671008 (NCT02659631)] in solid tumors, including triple‐negative breast cancer, head and neck cancer, and esophageal cancer.

## 5 Conclusions

In conclusion, our work establishes, for the first time, *CDH3*/P‐cadherin as a novel oncogenic molecule in GBM. We demonstrated that this cell adhesion molecule is overexpressed in a subset of high‐grade gliomas, and affects GBM aggressiveness *in vitro*. *In vivo*, P‐cadherin associates with increased tumor growth and shorter survival. In the clinical setting, *CDH3* correlates with cancer‐related signatures and associates with poor prognosis of GBM patients.

## Conflict of interest

The authors declare no conflict of interest.

## Author contributions

EPM, CSG, MP, RC, ASR, and VMG conducted experiments. EPM, CSG, MP, RC, ASR, RT, FB, NS, JP, and BMC analyzed data. FP, AAP, CC, and CCF provided patient samples. EPM and BMC wrote the manuscript. All authors reviewed and approved the manuscript.

### Peer Review

The peer review history for this article is available at https://publons.com/publon/10.1002/1878‐0261.13162.

## Supporting information


**Fig. S1**. P‐cadherin expression in glioma samples.
**Fig. S2**. Characterization of *CDH3* expression in 12 GBM cell models.
**Fig. S3**. P‐cadherin does not influence the expression of proliferation, anti‐apoptotic, and stemness markers in mice intracranial tumors.
**Fig. S4**. Stable P‐cadherin knockdown is associated with decreased aggressiveness features of GBML18 cells *in vitro*.
**Fig. S5**. Silencing of *CDH3* with distinct shRNA clones affects cancer hallmarks in GBML42, a GBM patient‐derived culture.
**Fig. S6**. *CDH3* influences the cell cycle of GBM cells.
**Fig. S7**. Molecular signatures associated with *CDH3*‐positively and *CDH3*‐inversely correlated genes in GBM patients from TCGA, and validation in GBM primary cultures.
**Table S1**. Sequence of primers used for qRT‐PCR studies.
**Table S2**. Information of antibodies used for western blot and immunohistochemistry.

## Data Availability

TCGA data used are publicly available (https://doi.org/10.1038/nature07385).
