# Supplementary material for: Cadherin‐3 is a novel oncogenic biomarker with prognostic value in glioblastoma
Source: Mol Oncol. 2022 Jun 10;16(14):2611–31. doi: 10.1002/1878-0261.13162 (PMC9297769; doi:10.1002/1878-0261.13162)
Supplement: Supplementary file 1 — Fig. S1. P‐cadherin expression in glioma samples. Fig. S2. Characterization of CDH3 expression in 12 GBM cell models. Fig. S3. P‐cadherin does not influence the expression of proliferation, anti‐apoptotic, and stemness markers in mice intracranial tumors. Fig. S4. Stable P‐cadherin knockdown is associated with decreased aggressiveness features of GBML18 cells in vitro. Fig. S5. Silencing of CDH3 with distinct shRNA clones affects cancer hallmarks in GBML42, a GBM patient‐derived culture. Fig. S6. CDH3 influences the cell cycle of GBM cells. Fig. S7. Molecular signatures associated with CDH3‐positively and CDH3‐inversely correlated genes in GBM patients from TCGA, and validation in GBM primary cultures. Table S1. Sequence of primers used for qRT‐PCR studies. Table S2. Information of antibodies used for western blot and immunohistochemistry. [file MOL2-16-2611-s001.pdf]

# *Cadherin-3* is a novel oncogenic biomarker with prognostic value in glioblastoma

Eduarda P. Martins, Céline S. Gonçalves, Marta Pojo, Rita Carvalho, Ana S. Ribeiro, Vera Miranda-Gonçalves, Ricardo Taipa, Fernando Pardal, Afonso A. Pinto, Carlos Custódia, Cláudia C. Faria, Fátima Baltazar, Nuno Sousa, Joana Paredes, Bruno M. Costa

## Supporting Information:

- Supplementary Tables
- Supplementary Figure Legends
- Supplementary Figures

## Supplementary tables

**Table S1.** Sequence of primers used for qRT-PCR studies.

| Gene | Sense primer | Antisense primer | Annealing temperature (° C) |
|---|---|---|---|
| *FZD4* | AGAACCTCGGCTACAACGTG | GGGTTCACAGCGTCTCTTGA | 60 |
| *PDGFRB* | GACACCAGCTCCGTCCTCTA | GGCTGTCACAGGAGATGGTT | 61 |
| *ZEB1* | TGCTCCCTGTGCAGTTACAC | TTACACCCAGACTGCGTCAC | 62 |
| *NANOG* | ATACCTCAGCCTCCAGCAGA | CTGGGGTAGGTAGGTGCTGA | 60 |
| *VIM* | GGGACCTCTACGAGGAGGAG | AAGATTGCAGGGTGTTTTCG | 60 |
| *TBP* | GAGCTGTGATGTGAAGTTTCC | TCTGGGTTTGATCATTCTGTAG | 60 |

**Table S2.** Information of antibodies used for western blot and immunohistochemistry.

| Antibody | Product reference | Technique | Incubation |
|---|---|---|---|
| CDH3/P-cadherin | 610227, clone 56, BD Biosciences | WB | 1:500, ON, 4 °C |
| | | IHC | 1:50, 1 h, RT |
| Ki-67 | 550609, BD Biosciences | IHC | 1:200, ON, 4 °C |
| Cyclin D1 | 2978, Cell Signaling | IHC | 1:100, ON, 4 °C |
| SOX2 | AB5603, EMD Millipore | IHC | 1:500, ON, 4 °C |
| NESTIN | MAB5326, EMD Millipore | IHC | 1:100, ON, 4 °C |
| BCL2 | 2870, Cell Signaling | IHC | 1:200, ON, 4 °C |
| α-Tubulin | T6199, Sigma | WB | 1:10000, 1 h, RT |
| HSC70 | sc-7298, Santa Cruz Biotechnology | WB | 1:8000, ON, 4 °C |
| mouse IgG - HRP conjugated | sc-2054, Santa Cruz Biotechnology | WB | 1:1000, 1 h, RT |

(WB: Western Blot; IHC: Immunohistochemistry; ON: Overnight; RT: Room Temperature).

## Supplementary figure legends

**Figure S1. P-cadherin expression in glioma samples.**

Representative images of P-cadherin IHC stainings in glioma samples from Hospital de Braga organized according to WHO glioma grades (II, III, and IV). WHO glioma grade II samples classified as negative for P-cadherin (images **a**, **b,** and **c**); WHO grade III P-cadherin-negative (image **d**) and -positive glioma samples (images **e** and **f**); grade IV P-cadherin-negative (image **g**) and -positive glioma samples (images **h** and **i**). Scale bars = 50 µm.

**Figure S2. Characterization of *CDH3* expression in 12 GBM cell models.**

*CDH3* expression assessed by qRT-PCR in six GBM cell lines (U87MG, U373MG, U251MG, SNB19, A172, and LN229) and six GBM patient-derived primary cultures (GBML18, GBML24, GBML26, GBML42, GBML45, and GBML95). Data is presented as mean ± SD.

**Figure S3. P-cadherin does not influence the expression of proliferation, anti-apoptotic, and stemness markers in mice intracranial tumors.**

**(A-B)** Representative images of the immunostainings of proliferation, anti-apoptotic and stemness markers in intracranial tumors formed upon the injection of U87-Ctrl or U87-CDH3 cells in NSG mice and collected at the endpoint of overall survival (inferior left corner was magnified; representative images are shown) **(A)**, and respective quantification **(B)**. Truncated violin plots indicate the percentage of area stained in IHC for the two distinct groups (dashed and dotted lines represent the median and quartiles, respectively; at least three animals per group were evaluated***;*** two-sided unpaired *t*-test; ns: not significant).

**Figure S4. Stable P-cadherin knockdown is associated with decreased aggressiveness features of GBML18 cells *in vitro*.**

**(A-B)** Confirmation of *CDH3*/P-cadherin silencing after lentiviral transduction in GBML18 patient-derived culture through qRT-PCR (n = 3; **A**) and western blot **(B)**. **(C-D)** Effect of P-cadherin in GBML18 cell viability silenced for *CDH3* and in their control counterpart, evaluated by trypan blue exclusion assay (n = 3; **C**) and MTS (n = 3; **D**). **(E-F)** Migration capacity was tested in P-cadherin-high and P-cadherin-low cells through wound healing assay (n = 4; representative images are shown for the two conditions **(E)**; scale bar = 500 µm). **(F)** Wound closure was followed and quantified for 24 hours. (Two-sided paired *t*-test was used, except for migration

assay in which a two-way ANOVA followed by post-hoc Sidak's test was applied; data is presented as mean ± SEM; ns: not significant; * $p < 0.05$; ** $p < 0.01$; *** $p < 0.001$).

**Figure S5. Silencing of *CDH3* with distinct shRNA clones affects cancer hallmarks in GBML42, a GBM patient-derived culture.**

**(A)** Success of knockdown of *CDH3* in GBML42 primary culture after transduction with two different shRNA clones was confirmed at the mRNA level (n = 3). **(B-C)** *CDH3* impact in GBML42 cell viability was tested by trypan blue (n = 4; **B**) and MTS (n = 3; **C**). **(D-E)** Wound healing assay was used to test the effect of *CDH3* in cell migration for up to 24 hours (n = 4; representative images are shown **(D)**; scale bar = 500 µm) and quantified as the percentage of wound closure **(E)**. (Two-sided paired *t*-test, except for migration assay in which a two-way ANOVA followed by post-hoc Sidak's test was applied; data is presented as mean ± SEM; ns: not significant; * $p < 0.05$; ** $p < 0.01$; *** $p < 0.001$).

**Figure S6. *CDH3* influences the cell cycle of GBM cells.**

**(A-B)** The impact of *CDH3* in the cell cycle was evaluated by flow cytometry using propidium iodide in GBML18 **(A)** and GBML42 **(B)** GBM-patient derived cultures with matching pairs of *CDH3*-high/-low cells. (Two-way ANOVA followed by post-hoc Sidak's test was applied; ns: not significant; * $p < 0.05$; ** $p < 0.01$; **** $p < 0.0001$).

**Figure S7. Molecular signatures associated with *CDH3*-positively and *CDH3*-inversely correlated genes in GBM patients from TCGA, and validation in GBM primary cultures.**

Enrichr was used to determine the enriched KEGG pathways **(A)**, and gene ontology molecular functions **(B)**, biological processes **(C)**, and cellular components **(D)** among the most *CDH3* correlated genes (Spearman r > 0.3 and r < -0.3). **(E)** qRT-PCR validation in *CDH3*-genetically manipulated primary GBM cells (GBML18-shCtrl *vs.* GBML18-shCDH3 and GBML42-shCtrl *vs.* GBML42-shCDH3-C) of key genes involved in relevant signaling pathways (*WNT1*, *WNT5A*, *FZD4* for WNT/β-catenin signaling; *PDGFRB* for PI3K-mTOR signaling) and biological processes (*NANOG* and *VIM* for stem capacity; *ZEB1* for cell motility) identified with *in silico* data from patients. (Two-sided unpaired *t*-test was used, data is presented as mean ± SD; u.d.: undetectable; ns: not significant; * $p < 0.05$; ** $p < 0.01$; *** $p < 0.001$).

# Figure S1

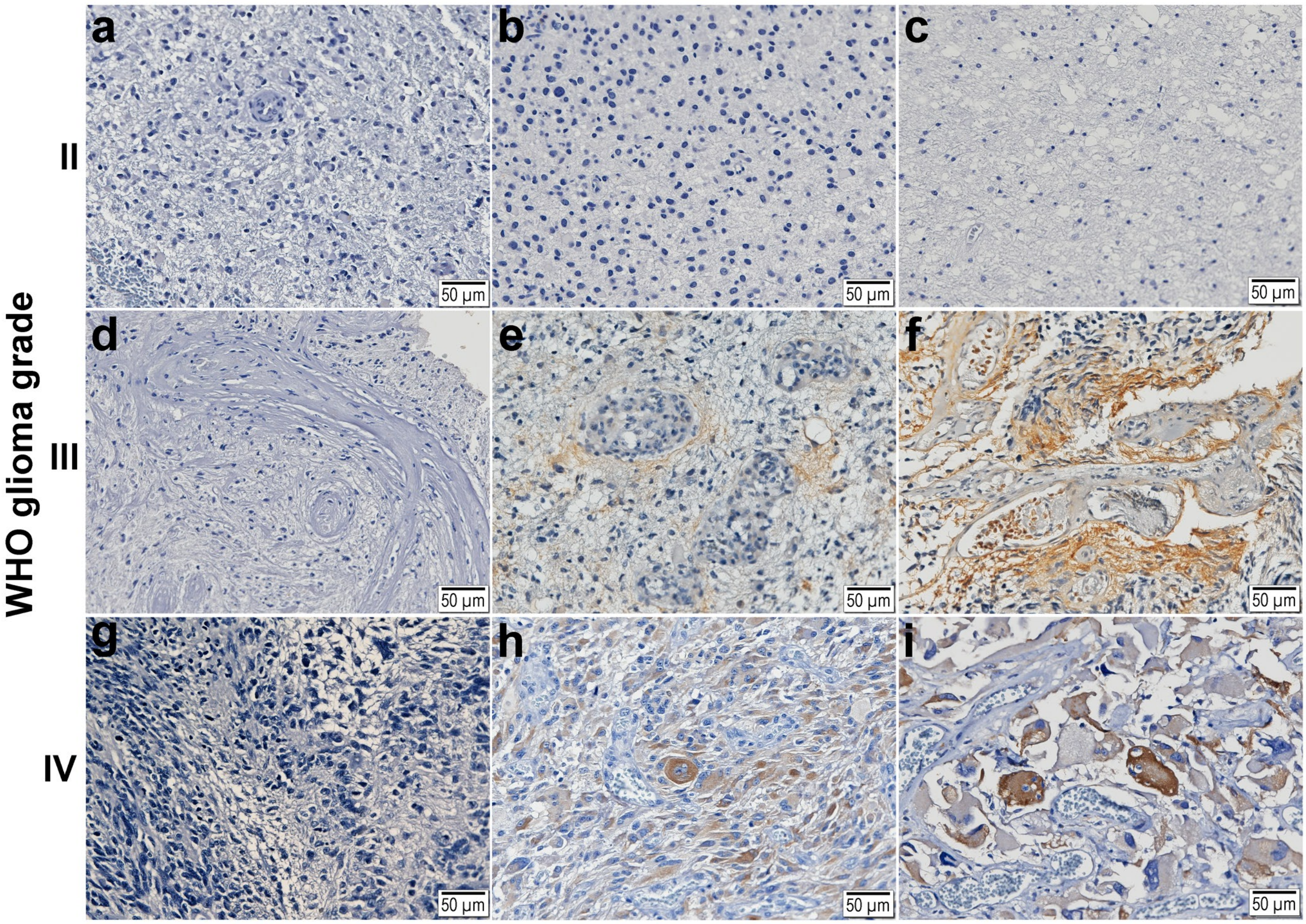

# Figure S2

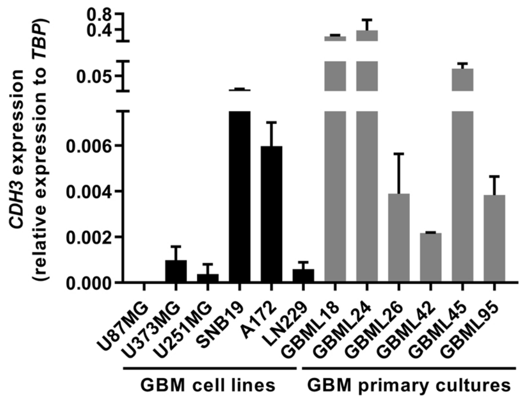
CDH3 expression
(relative expression to TBP)
0.8
0.4
0.05
0.006
0.004
0.002
0.000
U87MG
U373MG
U251MG
SNB19
A172
LN229
GBML18
GBML24
GBML26
GBML42
GBML45
GBML95
GBM cell lines
GBM primary cultures

# Figure S3

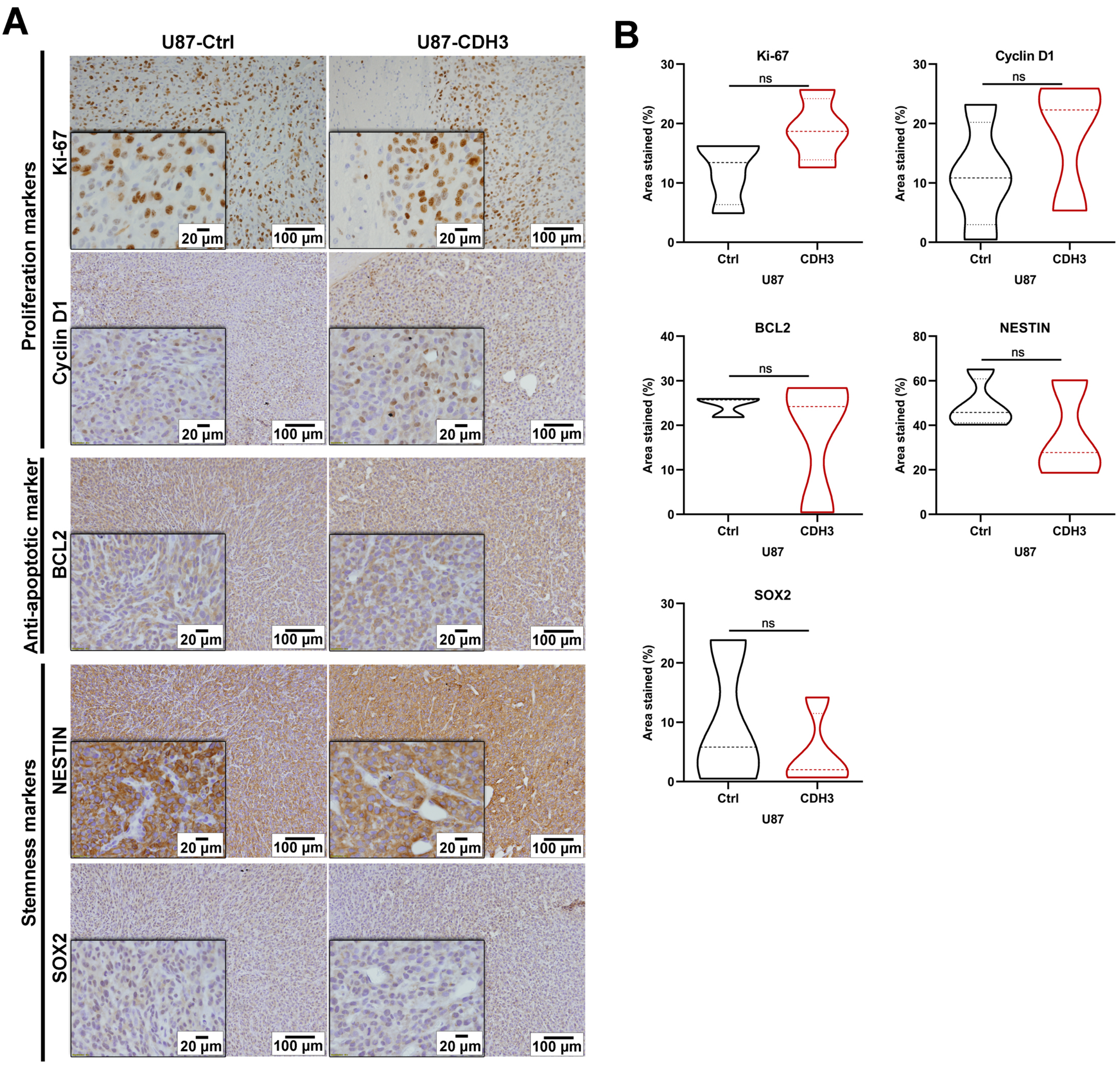
A
U87-Ctrl
U87-CDH3
Proliferation markers
Ki-67
Cyclin D1
Anti-apoptotic marker
BCL2
Stemness markers
NESTIN
SOX2
20 µm
100 µm
B
Ki-67
Cyclin D1
BCL2
NESTIN
SOX2
ns
Area stained (%)
Ctrl
CDH3
U87

# Figure S4

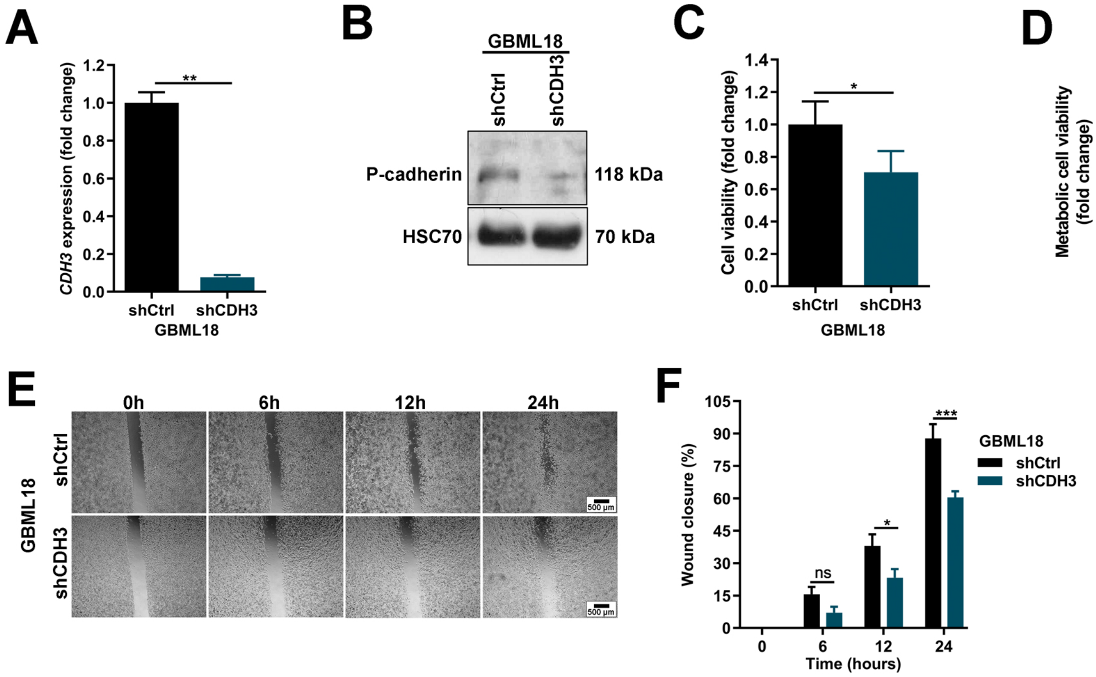

A
CDH3 expression (fold change)
**
shCtrl
shCDH3
GBML18
B
GBML18
shCtrl
shCDH3
P-cadherin
118 kDa
HSC70
70 kDa
C
Cell viability (fold change)
*
shCtrl
shCDH3
GBML18
D
Metabolic cell viability (fold change)
*
shCtrl
shCDH3
GBML18
E
0h
6h
12h
24h
GBML18
shCtrl
shCDH3
500 µm
F
Wound closure (%)
Time (hours)
ns
*
***
GBML18
shCtrl
shCDH3

# Figure S5

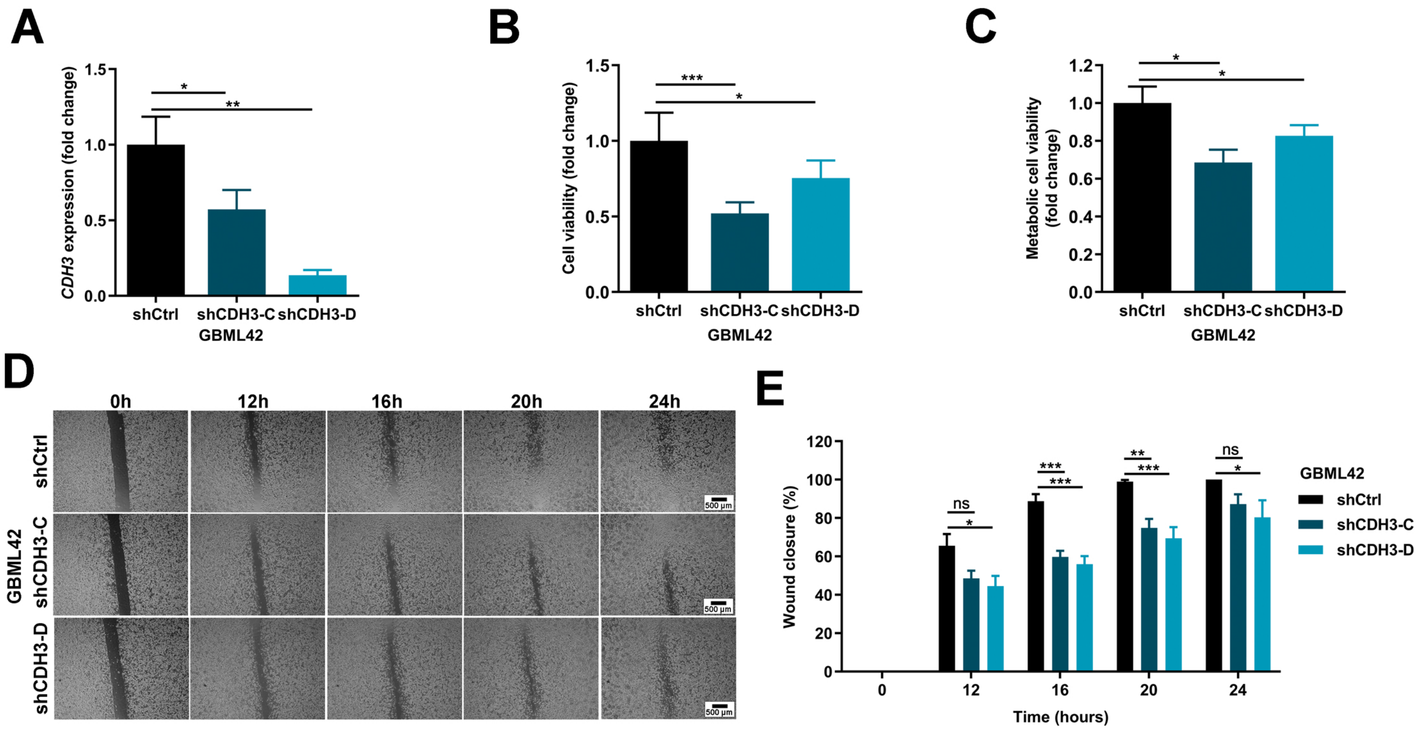

A
CDH3 expression (fold change)
1.5
1.0
0.5
0.0
*
**
shCtrl
shCDH3-C
shCDH3-D
GBML42
B
Cell viability (fold change)
***
*
GBML42
C
Metabolic cell viability (fold change)
1.2
0.8
0.6
0.4
0.2
GBML42
D
0h
12h
16h
20h
24h
shCtrl
GBML42 shCDH3-C
shCDH3-D
500 µm
E
Wound closure (%)
120
100
80
60
40
20
0
12
16
20
24
Time (hours)
ns
GBML42
shCtrl
shCDH3-C
shCDH3-D

# Figure S6

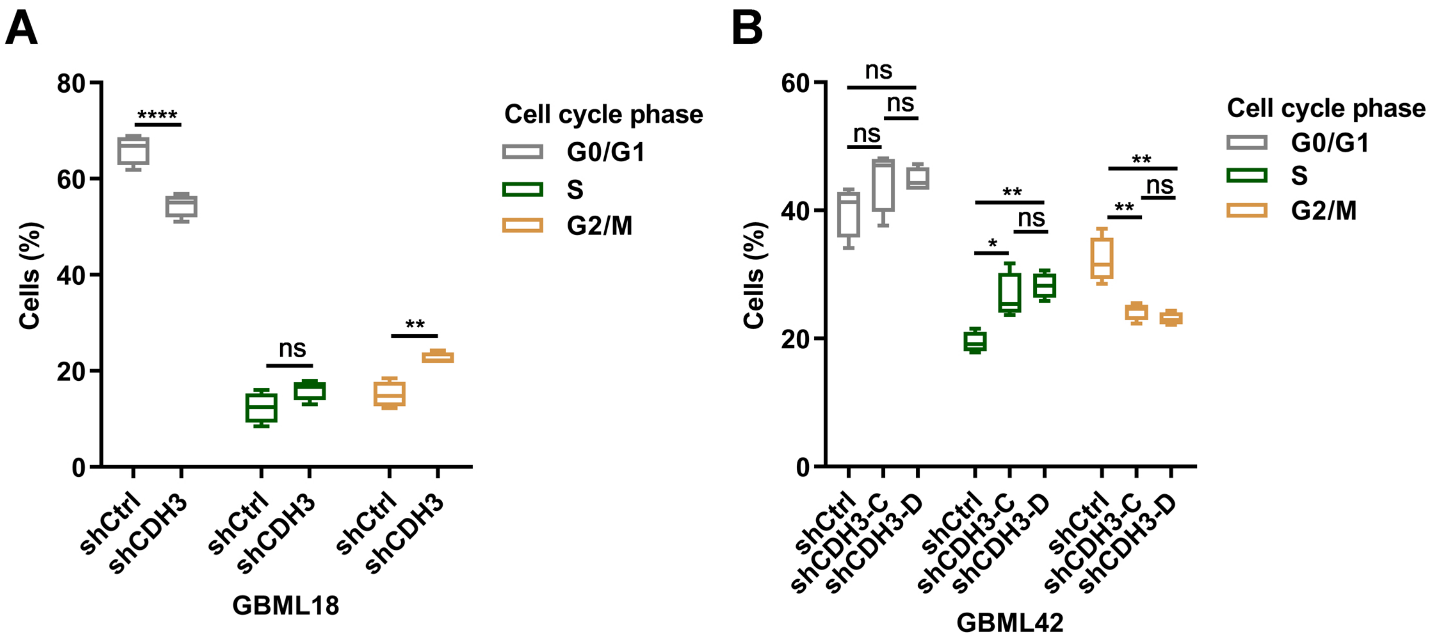
A
B
Cells (%)
0
20
40
60
80
****
ns
**
shCtrl
shCDH3
GBML18
Cell cycle phase
G0/G1
S
G2/M
shCDH3-C
shCDH3-D
*
GBML42

# Figure S7

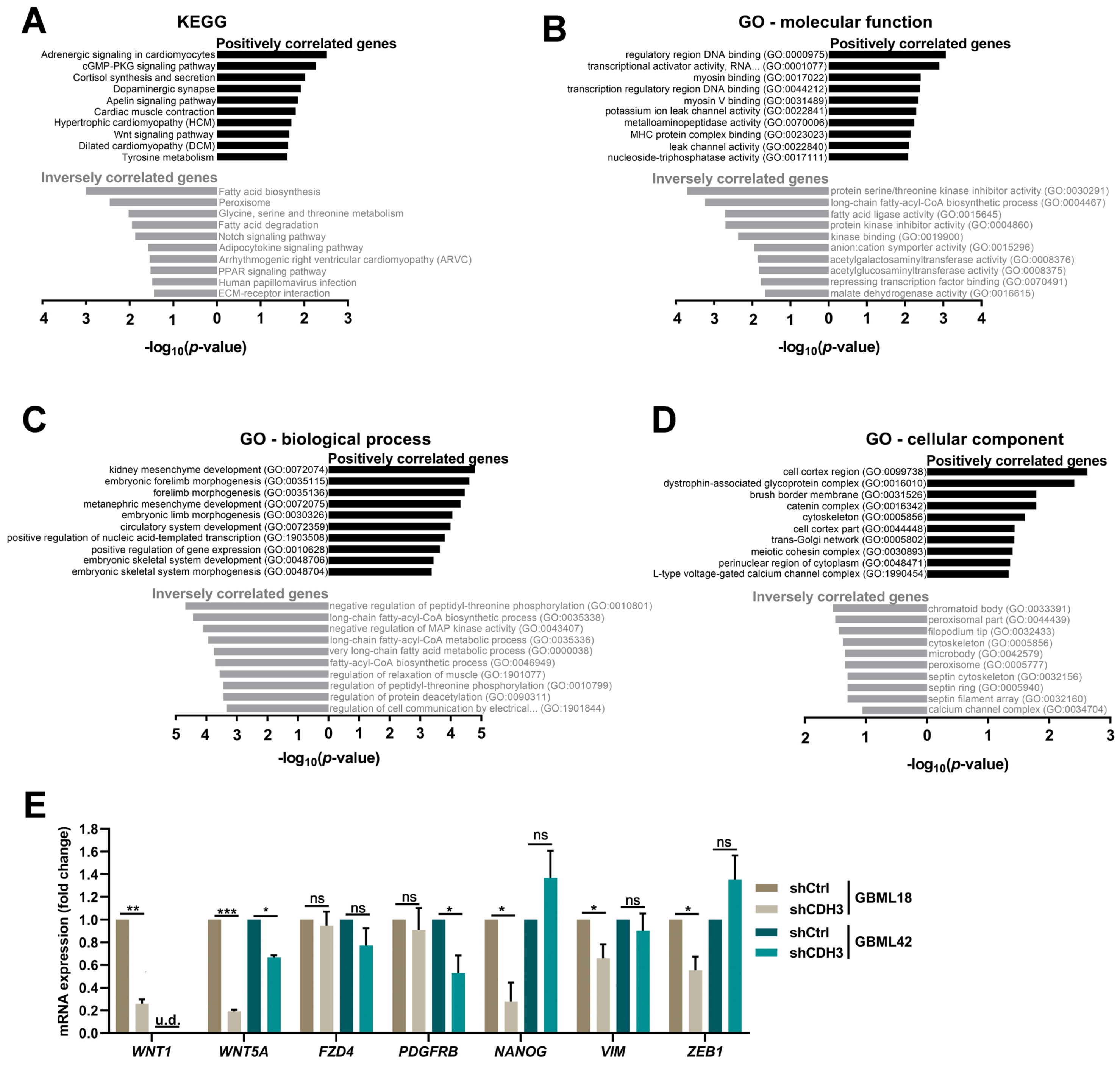

A
KEGG
Positively correlated genes
Adrenergic signaling in cardiomyocytes
cGMP-PKG signaling pathway
Cortisol synthesis and secretion
Dopaminergic synapse
Apelin signaling pathway
Cardiac muscle contraction
Hypertrophic cardiomyopathy (HCM)
Wnt signaling pathway
Dilated cardiomyopathy (DCM)
Tyrosine metabolism
Inversely correlated genes
Fatty acid biosynthesis
Peroxisome
Glycine, serine and threonine metabolism
Fatty acid degradation
Notch signaling pathway
Adipocytokine signaling pathway
Arrhythmogenic right ventricular cardiomyopathy (ARVC)
PPAR signaling pathway
Human papillomavirus infection
ECM-receptor interaction
-log10(p-value)
B
GO - molecular function
Positively correlated genes
regulatory region DNA binding (GO:0000975)
transcriptional activator activity, RNA... (GO:0001077)
myosin binding (GO:0017022)
transcription regulatory region DNA binding (GO:0044212)
myosin V binding (GO:0031489)
potassium ion leak channel activity (GO:0022841)
metalloaminopeptidase activity (GO:0070006)
MHC protein complex binding (GO:0023023)
leak channel activity (GO:0022840)
nucleoside-triphosphatase activity (GO:0017111)
Inversely correlated genes
protein serine/threonine kinase inhibitor activity (GO:0030291)
long-chain fatty-acyl-CoA biosynthetic process (GO:0004467)
fatty acid ligase activity (GO:0015645)
protein kinase inhibitor activity (GO:0004860)
kinase binding (GO:0019900)
anion:cation symporter activity (GO:0015296)
acetylgalactosaminyltransferase activity (GO:0008376)
acetylglucosaminyltransferase activity (GO:0008375)
repressing transcription factor binding (GO:0070491)
malate dehydrogenase activity (GO:0016615)
-log10(p-value)
C
GO - biological process
Positively correlated genes
kidney mesenchyme development (GO:0072074)
embryonic forelimb morphogenesis (GO:0035115)
forelimb morphogenesis (GO:0035136)
metanephric mesenchyme development (GO:0072075)
embryonic limb morphogenesis (GO:0030326)
circulatory system development (GO:0072359)
positive regulation of nucleic acid-templated transcription (GO:1903508)
positive regulation of gene expression (GO:0010628)
embryonic skeletal system development (GO:0048706)
embryonic skeletal system morphogenesis (GO:0048704)
Inversely correlated genes
negative regulation of peptidyl-threonine phosphorylation (GO:0010801)
long-chain fatty-acyl-CoA biosynthetic process (GO:0035338)
negative regulation of MAP kinase activity (GO:0043407)
long-chain fatty-acyl-CoA metabolic process (GO:0035336)
very long-chain fatty acid metabolic process (GO:0000038)
fatty-acyl-CoA biosynthetic process (GO:0046949)
regulation of relaxation of muscle (GO:1901077)
regulation of peptidyl-threonine phosphorylation (GO:0010799)
regulation of protein deacetylation (GO:0090311)
regulation of cell communication by electrical... (GO:1901844)
-log10(p-value)
D
GO - cellular component
Positively correlated genes
cell cortex region (GO:0099738)
dystrophin-associated glycoprotein complex (GO:0016010)
brush border membrane (GO:0031526)
catenin complex (GO:0016342)
cytoskeleton (GO:0005856)
cell cortex part (GO:0044448)
trans-Golgi network (GO:0005802)
meiotic cohesin complex (GO:0030893)
perinuclear region of cytoplasm (GO:0048471)
L-type voltage-gated calcium channel complex (GO:1990454)
Inversely correlated genes
chromatoid body (GO:0033391)
peroxisomal part (GO:0044439)
filopodium tip (GO:0032433)
cytoskeleton (GO:0005856)
microbody (GO:0042579)
peroxisome (GO:0005777)
septin cytoskeleton (GO:0032156)
septin ring (GO:0005940)
septin filament array (GO:0032160)
calcium channel complex (GO:0034704)
-log10(p-value)
E
mRNA expression (fold change)
WNT1
WNT5A
FZD4
PDGFRB
NANOG
VIM
ZEB1
u.d.
shCtrl
shCDH3
GBML18
shCtrl
shCDH3
GBML42
